# Intronless WNT10B-short variant underlies new recurrent allele-specific rearrangement in acute myeloid leukaemia

**DOI:** 10.1038/srep37201

**Published:** 2016-11-17

**Authors:** Francesca Lazzaroni, Luca Del Giacco, Daniele Biasci, Mauro Turrini, Laura Prosperi, Roberto Brusamolino, Roberto Cairoli, Alessandro Beghini

**Affiliations:** 1 Department of Health Sciences, University of Milan, Milan, Italy; 2 Department of Biosciences, University of Milan, Milan, Italy; 3 Cambridge Institute for Medical Research, University of Cambridge, Cambridge, UK; 4 Department of Internal Medicine, Valduce Hospital, Como, Italy; 5 Department of Pathology, Niguarda Hospital, Milan, Italy; 6 Department of Oncology, Hematology Unit, Niguarda Hospital, Milan, Italy

## Abstract

Defects in the control of Wnt signaling have emerged as a recurrent mechanism involved in cancer pathogenesis and acute myeloid leukaemia (AML), including the hematopoietic regeneration-associated WNT10B in AC133^bright^ leukaemia cells, although the existence of a specific mechanism remains unproven. We have obtained evidences for a recurrent rearrangement, which involved the WNT10B locus (WNT10B^R^) within intron 1 (IVS1) and flanked at the 5′ by non-human sequences whose origin remains to be elucidated; it also expressed a transcript variant (WNT10B^IVS1^) which was mainly detected in a cohort of patients with intermediate/unfavorable risk AML. We also identified in two separate cases, affected by AML and breast cancer respectively, a genomic transposable short form of human WNT10B (ht-WNT10B). The intronless ht-WNT10B resembles a long non-coding RNA (lncRNA), which suggests its involvement in a non-random microhomology-mediated recombination generating the rearranged WNT10B^R^. Furthermore, our studies supports an autocrine activation primed by the formation of WNT10B-FZD4/5 complexes in the breast cancer MCF7 cells that express the WNT10B^IVS1^. Chemical interference of WNT-ligands production by the porcupine inhibitor IWP-2 achieved a dose-dependent suppression of the WNT10B-FZD4/5 interactions. These results present the first evidence for a recurrent rearrangement promoted by a mobile ht-WNT10B oncogene, as a relevant mechanism for Wnt involvement in human cancer.

A longstanding question in cancer biology has been the nature of mechanisms that control initiation and growth of a hierarchically organized population of cells sustained by cancer stem cells (CSCs) at their apex^1^. It is well established that canonical Wnt signalling plays a major role in fuelling stem cell activity and sustaining tissue regeneration^2^,^3^ in a dose dependent manner in several adult stem cell niches including bone marrow^4^,^5^,^6^ and mammary gland^7^. Wnt proteins have also been extensively studied in connection with malignancy^8^,^9^ and they are causatively involved in the development of several types of leukaemias^10^,^11^,^12^,^13^,^14^,^15^. Nontheless, it remains unclear both what molecular features govern the specific functional properties of leukaemia initiating cells (LIC) and which genome-destabilizing processes promote leukaemia stemness^16^.

Recent insights into regenerative processes point to WNT10B as a candidate potentially linking tissue regeneration and cancer^2^,^17^,^18^,^19^. We recently reported evidence supporting the influence of the haematopoietic regeneration-associated WNT10B on AC133^bright^ cells in human AML via an autocrine/paracrine loop^20^.

## Results and Discussion

### Transient *wnt10b* overexpression in zebrafish embryos promotes the accumulation of haematopoietic precursors

**Figure 1:**
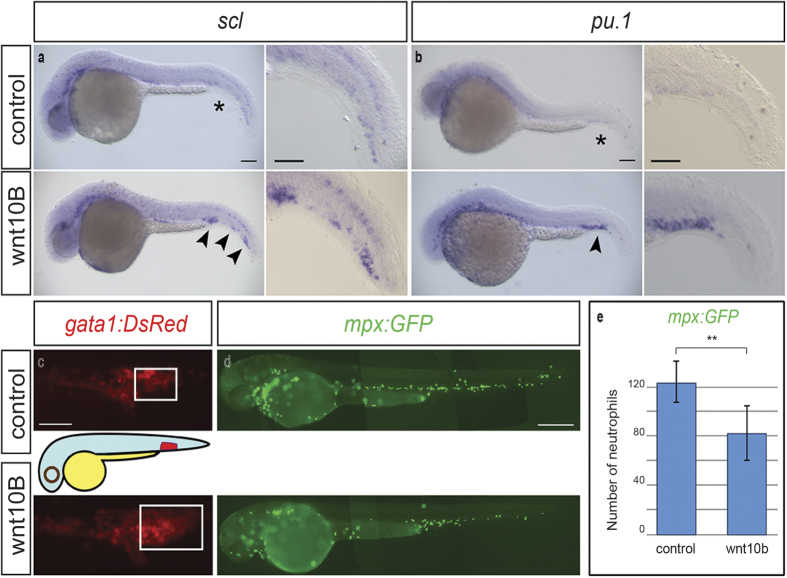
*wnt10b* overexpression in zebrafish embryos. (**a**,**b**) Representative whole mount *in-situ* hybridizations to *scl* (**a**) and *pu*.*1* (**b**) on 28 hours post fertilization (hpf) embryos. Increased number of cells expressing the two genes in the PBI of *wnt10b*-injected embryos (arrowheads; *scl*, n = 62; *pu*.*1*, n = 114) in comparison to controls (asterisks; *scl*, n = 34; *pu*.*1*, n = 66). Scale bar, 100 μm. Magnification scale bar, 50 μm. (**c**) Forced *wnt10b* upregulation in 28 hpf *gata1:*DsRed transgenic embryos, expressing the red fluorescent protein DsRed in erythroid progenitors, caused the increase in the number of *gata1*-positive cells in the PBI in 30% of the injected embryos (*wnt10b*-injected embryos n = 58 and controls n = 41). Scale bar, 125 μm. (**d**) Representative *wnt10b*-injected and control 48 hpf *mpx:*GFP transgenic embryos characterized by GFP expression in differentiated neutrophils. Scale bar, 300 μm. (**e**) *wnt10b* overexpression in 48 hpf *mpx:*GFP transgenic embryos caused a marked reduction in the number of GFP-positive circulating cells in 52% of the injected embryos (*wnt10b*-injected embryos, n = 18; control embryos, n = 10, mean ± s.d.). **<0.01 with Student’s *t*-test.

To asses if the reported WNT10B-overexpression in the hematopoietic microenvironment could be an important perturbing factor of myeloid cells and a possible adjuvant in AML development, synthetic mRNA was microinjected in the zebrafish zygote in order to determine the effects of transient *wnt10b* overexpression on early haematopoiesis. This was performed to mimic, *in vivo*, the Wnt pathway aberrant deregulation observed in the leukaemia bone marrow micro-environment. The injected embryos displayed developmental defects, such as expansion of the ventral tissues, dorsalization, and lack of the anterior-most cephalic regions, which was to be expected from the excessive activation of the Wnt pathway^21^. Remarkably, we also observed the expansion of the posterior blood island (PBI), and the caudal-most haematopoetic tissue hosting erythromyeloid progenitors, which suggest some effect on haematopoiesis (data not shown). We therefore focused on the consequences of *wnt10b* upregulation by analyzing the expression of *scl* (Fig. 1a), *pu*.*1* (Fig. 1b), and *gata1* (Fig. 1c), which are, respectively, distinctive markers of erythromyeloid, myeloid, and erythroid progenitors^22^. Our results show that elevated *wnt10b* expression caused the expansion of these cell populations (Fig. 1a–c and Supplementary Fig. 1a–d). While the number of progenitors increased, that of circulating neutrophils (Fig. 1d) reduced markedly (Fig. 1e) in the transgenic line expressing GFP under the neutrophil-specific myeloperoxidase (*mpx*) promoter. These findings indicate that *wnt10b* overexpression promotes the accumulation of haematopoietic precursors at the expense of more differentiated cell types, resembling conditions, such as pre-leukemic expansion of haematopoietic stem cell (HSC), which are characterized by an imbalance between immature and differentiated blood cells^23^. Taken together, these results support a critical role for *wnt10b* in haematopoietic progenitors homeostasis.

### Identification of recurrent rearrangement generating the WNT10B^R^ allele

**Figure 2:**
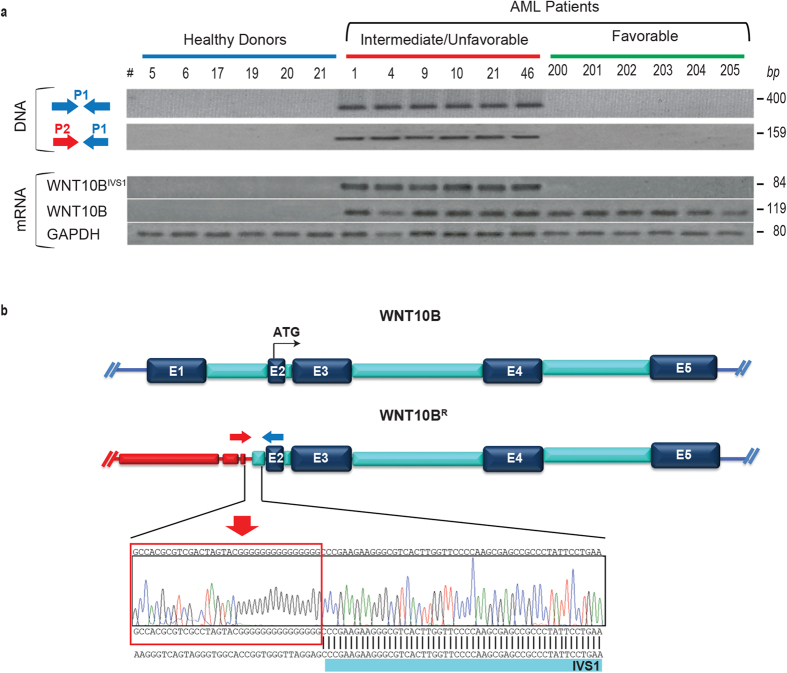
WNT10B^R^ identification in AML patients. (**a**) Genomic identification (P1 single primer extension, upper panel) performed on MNC cells in a representative cohort of n = 6 healthy donors and n = 12 AML patients. In the lower panel the WNT10B and WNT10B^IVS1^ gene expression analysis with the GAPDH as housekeeping gene. (**b**) Schematic diagram of genomic WNT10B locus, consisting in five exons (upper panel). In the lower panel is represented a scheme of the rearrangement involving the WNT10B locus upstream the exon 2, defining the sequence at WNT10B 5′-end flanking region. The electropherogram shows the WNT10B^R^ sequence and the red line squared non-human sequence extending upstream the portion of IVS1 (AML#9).

To assess a possible involvement of a key genetic event behind the deregulated WNT10B expression observed in AML, we first performed on leukemic bone marrow mononuclear cells (BMNCs), a single-primer extension/amplification of the 5′ flanking sequences upstream of exon 2 which contains the starting codon of WNT10B (Fig. 2a), in a representative cohort of Italian AML patients and healthy donors (Supplementary Table 1).

A single amplification product of 400 bp was observed in genomic DNA (Fig. 2a) in all intermediate-adverse risk AML patients; subsequent sequence analysis revealed the insertion of a non-human DNA segment located 77 nucleotides upstream the end of IVS1 after a short G-rich stretch (Fig. 2b). The origin of the non-human DNA extending to the 5′ of WNT10B^R^ locus (Fig. 2b) could not be identified, but it resembles transposable DNA.

A 5′ RACE and WNT10B-alleles specific expression analysis were performed on the same cohort of patients in order to test for the presence of an expressed form derived from the rearranged allele observed in genomic DNA within IVS1. As previously observed, the normal WNT10B allele was expressed in all AML patients but not in BMNCs of healthy donors^20^. Conversely, the rearranged form, hereafter named WNT10B^IVS1^, was expressed exclusively in patients harboring the corresponding rearrangement in the genomic DNA (Fig. 2a). Analysis performed on the AC133-positive leukemic cell fraction reported the same results observed on the whole MNCs.

### WNT10B/WNT10B^IVS1^ expression ratio match with WHO risk stratification

**Figure 3:**
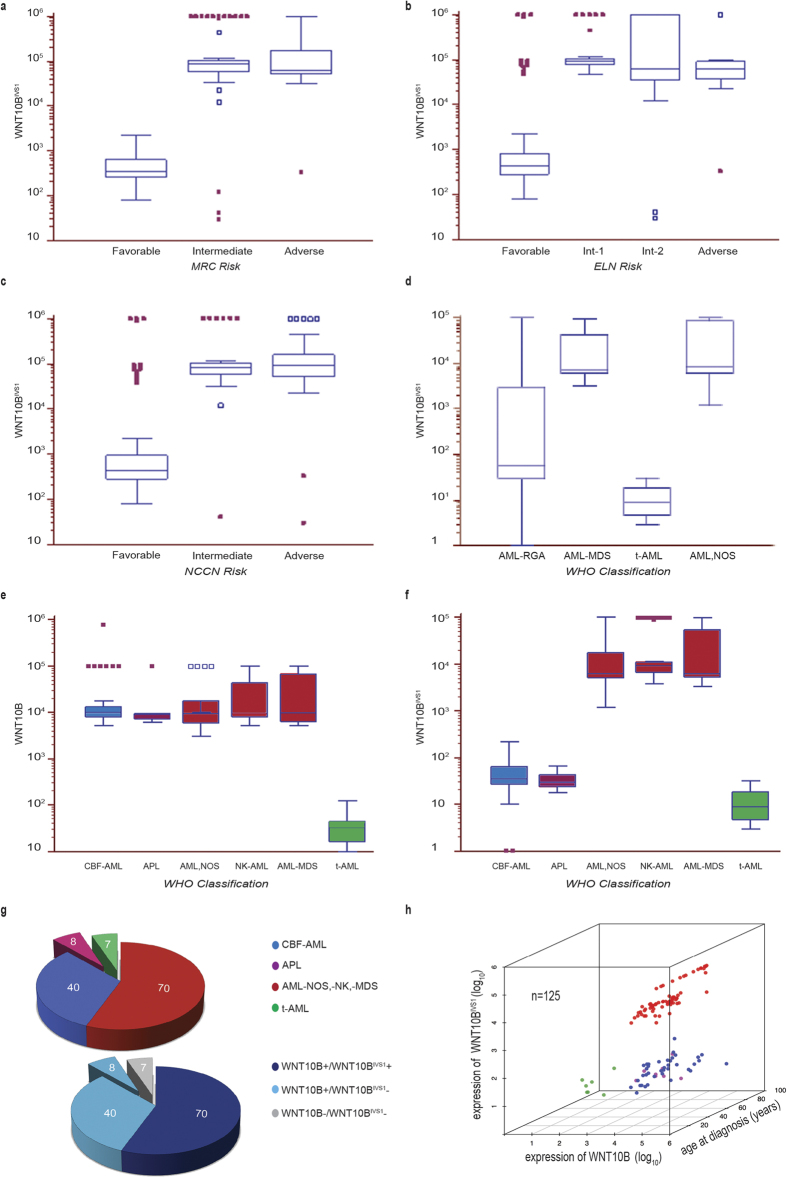
Box-plot distribution of the WNT10B/WNT10B^IVS1^ transcripts per AML classes. WNT10B^IVS1^ transcript levels showed a significative difference (*p* < 0.0001; Kruskal-Wallis test) between MRC (**a**), ELN (**b**) and NCNN (**c**) risk classes classification in a cohort of n = 125 AML patients (n = 70 intermediate and adverse-risk, n = 40 favorable-risk, n = 8 APL and n = 7 therapy-related). Comparative Mann-Whitney U-test showed a significative reduction of WNT10B^IVS1^ levels in favorable *versus* adverse-intermediate risk patients (*p* < 0.001 for all three classification system). No statistical significance in WNT10B^IVS1^ levels between intermediate and adverse-risk groups (MRC: Int *vs* Adv: *p* 0.4864; ELN: Int-1 *vs* Int-2 *p* 0.1500 and Int-1/2 *vs* Adv: *p* 0.6963; NCCN: Int *vs* Adv: *p* 0.9940). (**d**) Kruskal-Wallis test of WNT10B^IVS1^ transcript distribution per WHO classes, showed a significative difference in WNT10B^IVS1^ distribution (*p* < 0.0001). Comparative Mann-Whitney U-test: t-AML *vs* AML-RGA, AML-MDS and AML,NOS groups (*p* < 0.0005), AML-RGA *vs* t-AML, AML-MDS and AML,NOS groups (*p* < 0.003) and AML-MDS *vs* AML,NOS: p 0.5475. (**e**) Significative difference in WNT10B transcript levels between the distinct WHO splitted classes (*p* 0.0008; Kruskal-Wallis test), and comparative Mann-Whitney U-test endorsed a significative reduction in patients with therapy-related disease (*p* < 0.01). No statistical significance in WNT10B levels between all the other groups. (**f**) Significative difference in WNT10B^IVS1^ transcript levels between the distinct WHO splitted classes (*p* < 0.0001; Kruskal-Wallis test). Comparative Mann-Whitney U-test confirmed a significative reduction of WNT10B^IVS1^ levels in CBF-AML, APL, and therapy-related AML compared with patients with recurrent genetic abnormalities, NPM1/CEBPA mutation, MDS-related features, normal karyotype or other non-recurrent genetic number or structural abnormalities (*p* < 0.01). Data represent mean values ± s.d. (**g**) Graphical representation of patients cohort distribution (upper panel) and WNT10B/WNT10B^IVS1^ mRNAs classification of AML patients (lower panel). (**h**) Consensus clustering 3D scatter plot of WNT10B and WNT10B^IVS1^ transcript levels in the cohort of AML patients. Data represent mean values ± s.d. AML-RGA: AML with recurrent genetic abnormalities, AML-MDS: AML with myelodysplasia-related features, t-AML: therapy-related AML, AML,NOS: AML, not otherwise specified.

In order to determine the prevalence of the genomic WNT10B^R^ allele, we retrospectively investigated the WNT10B pattern of rearrangement acquisition on mononuclear cells from an Italian cohort of 125 AML patients (Supplementary Table 1 and Supplementary Fig. 2a–f). We analyzed the relative expression of the two transcript variants WNT10B and WNT10B^IVS1^ by Droplet Digital™ PCR (ddPCR) in the leukaemic patient population classified according to the scoring system of the Medical Research Council (MRC), the European LeukemiaNet (ELN), and the National Comprehensive Cancer Network (NCCN) scoring systems (Supplementary Figs 3a–f, and 5a–f). WNT10B was highly expressed in all groups, while WNT10B^IVS1^ expression was associated only with the intermediate/unfavourable-risk groups (Fig. 3a–d, and Supplementary Fig. 4a–d). Samples from individuals who developed a therapy-related AML (t-AML) were negative for both WNT10B (*p* < 0.002, Fig. 3e and Supplementary Fig. 4d,e) and WNT10B^IVS1^ alleles (*p* < 0.0005, Fig. 3d,f and Supplementary Fig. 4,f). These results therefore, evidenced a WNT10B^R^ frequency of 56% in the present AML cohort, and link the WNT10B/WNT10B^IVS1^ expression pattern to the WHO risk stratification greatly strengthening the idea that this new genetic abnormality against WNT10B locus may have a role supporting the leukemic clone in clinical context without recurrent cytogenetic abnormalities (Fig. 3g,h). Furthermore, statistical analysis showed no significative difference for both WNT10B and WNT10B^IVS1^ levels according to FLT3-ITD (*p* 0.8818 and *p* 0.1271 respectively; Kruskal-Wallis test) and to KIT mutational status (*p* 0.4561 and *p* 0.4871 respectively; Kruskal-Wallis test).

**Figure 4:**
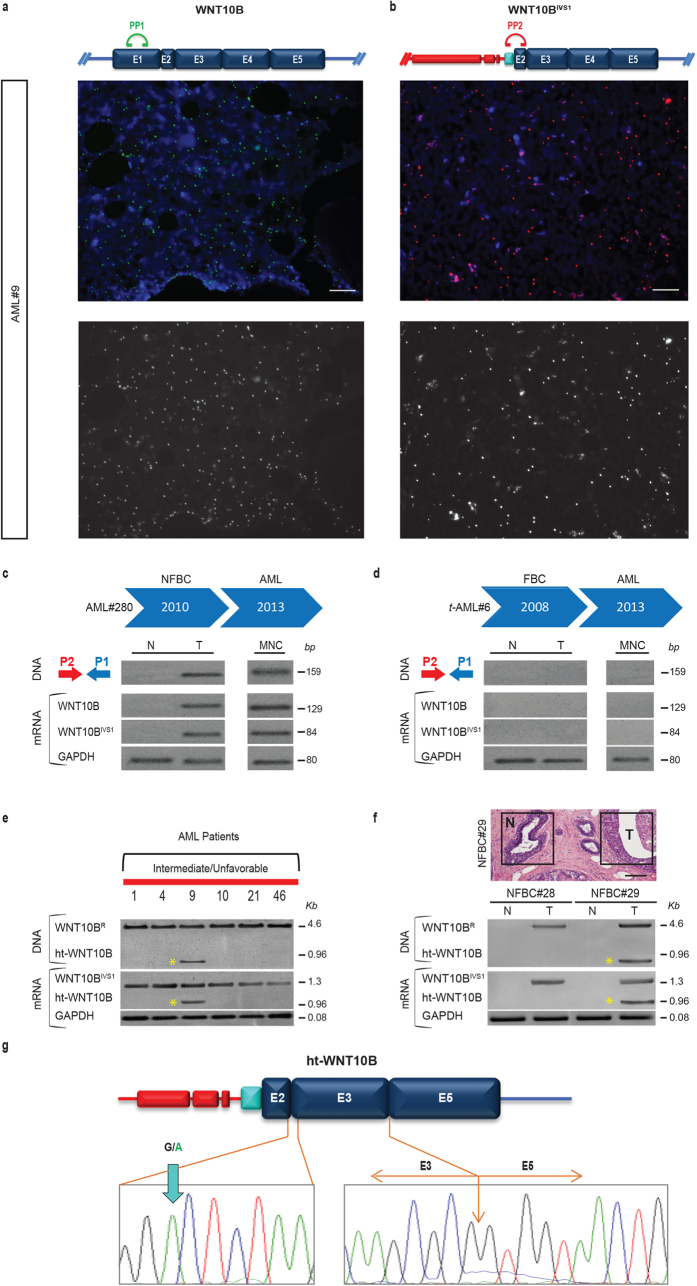
*In situ* detection of WNT10B^IVS1^ transcripts on AML bone marrow sections and identification of the ht*-*WNT10B. (**a**,**b**) Schematic representation of PP-1 and PP-2 padlock probes localized on WNT10B (**a**) and WNT10B^IVS1^ (**b**) transcripts. mRNA *in situ* detection of WNT10B (**a**) and WNT10B^IVS1^ (**b**) transcripts on AML#9 FFPE bone marrow consecutive sections with padlock probe and target-primed RCA. Hoechst staining for cell nuclei, Cy5 for WNT10B and Cy3 for WNT10B^IVS1^. In the lower panels the splitted grey scale images to enhance the RCPs. Maximum-Intensity Projection (MIP) images acquired with X20 magnification. Exposure times: DAPI 550 ms, Cy3 500 ms and Cy5 650 ms. Scale bar 20 μm. (**c**) The t-AML patient after radiation treatment (AML#280) is characterized by the presence of WNT10B^R^ allele at genomic level and its expression (WNT10B^IVS1^) is detected only in breast cancer tissue (T) and not in peritumoral normal area (N). AML sample at diagnosis resulted positive for WNT10B^R^ allele. (**d**) The single case of Familial Breast Cancer patient, evolved in t-AML (t-AML#6) after chemotherapy treatment, resulted negative for the presence of the rearranged WNT10B^R^ allele and for WNT10B-WNT10B^IVS1^ expression both in breast cancer and AML samples. (**e**,**f**) WNT10B analysis by PCR (P2-P10 primers) at genomic and mRNA levels in the same cohort of n = 6 Intermediate-Unfavorable risk AML patients (**e**) represented in Fig. 1 and in n = 2 NFBC patients. (**f**). Detection of rearranged WNT10B^R^ allele in all AML patients and in tumor tissues of NFBC patients (NFBC#28 and #29) an extra fragment of 0.96 kb (ht*-*WNT10B, asterisks) in the patient’s sample AML#9 (**e**) and in NFBC#29 (**f**). The RT-PCR analysis showed that these two form of WNT10B alleles are expressed. Top-right (**f**) brightfield microscopy photomicrograph of haematoxylin and eosin stained section of NFBC#29. Scale bar 50 μm. (**g**) Schematic representation of ht*-*WNT10B mobile element. The electropherogram of sequence analysis shows the nucleotide variation (G > A) in the exon 3, and the junction between exon 3 and exon 5 showing lacking of the exon 4. GAPDH, housekeeping gene. MNC, mononuclear cells. The expected MW (bp) of the amplicons are shown on the right side of each panel.

We demonstrated transcripts *in situ* with specific padlock probes and target-primed rolling-circle amplification (RCA) in order to assess the distribution and localization of the WNT10B/WNT10B^IVS1^ mRNAs in AML bone marrow sections using an independent, non-PCR-based technique. Reassuringly, we obtained abundant signals from both transcripts in bone marrow sections from two independent AML patients, visualizing both transcriptional variants with similar detection efficiency (Fig. 4a,b and Supplementary Fig. 5).

Two prototypic cases (t-AML#6, AML#280, Supplementary Table 2) were found within the leukemia patients in this study who were diagnosed with t-AML after a personal history of breast cancer treated with chemotherapy (CMT) or radiation therapy (XRT) respectively. We investigated both the presence of the rearranged WNT10B^R^ allele and the expression of WNT10B^IVS1^ in breast cancer tissues (T) and surrounding normal tissues (N) as well as in leukemia blasts (MNC) sampled at diagnosis (Fig. 4c,d).

In the case treated with XRT alone (AML#280), we could detect the presence of both the genomic WNT10B^R^ allele and the expression of the related WNT10B^IVS1^ transcript in non-familial breast cancer (NFBC) tissue, but not in normal surrounding tissues, which was present also in the secondary AML sample at diagnosis three years later (Fig. 4c).

In contrast, the case affected by familial breast cancer (FBC) resulted negative for the presence of the WNT10B^R^ genomic allele both in the breast cancer specimen and in the t-AML, which developed five years later following CMT (Fig. 4d).

These results were of particular interest considering recent observations that suggest AML diagnosed in patients after receiving XRT alone differ from t-AML occurring after CMT, and share genetic features and clinical behaviours with de novo AML^24^.

### Identification of the intronless short form ht*-*WNT10B

Recent results in breast cancer research support the rationale that the WNT10B/β-catenin/HMGA2 axis has a functional role in highly aggressive triple-negative breast cancers (TNBC) in humans^19^. This led us to assume an integration event consistent with MMTV-viral activation of the *Wnt-10b* locus in mice, and to link its overexpression to a high transforming potential in the mammary gland^25^. We therefore analyzed the coding region of WNT10B^R^ allele to assess the origin of the observed site-specific integration mechanism.

Amplification of the entire coding region using a primer pair specifically designed to detect the WNT10B^R^ allele shows the expected 4,6-kb fragment in the samples we had previously shown to harbor the presence of the abnormal rearrangement (intermediate-unfavorable risk patients) (Fig. 4e).

In addition to this fragment, that represents the rearranged allele, an extra amplicon was observed in the lane for the patient sample AML#9 (Fig. 4e). Sequence analysis of this extra band revealed the existence on the genomic level of an intronless WNT10B oncogene short form, hereafter named human transposable-WNT10B (ht-WNT10B). Specific RT-PCR analysis showed that ht-WNT10B is currently being expressed (Fig. 4e lower panel). Additionally, in the course of screening an Italian cohort of 35 de novo breast cancer patients (manuscript in preparation) we detected the same ht-WNT10B genetic element in one independent non-familial breast cancer patient (NFBC#29, Fig. 4f, and Supplementary Table 3).

Furthermore, in both cases in which ht-WNT10B was isolated, the sequence analysis revealed the absence of exons 1 and 4, and the presence of a G/A single nucleotide variation at the junction of exons 2 and 3 flanked at 5′ by the same 77 IVS1 nucleotides followed by non-human DNA as observed in WNT10B^R^ (Fig. 4g). This genetic element, resembling a long noncoding RNA (lncRNAs)^26^,^27^, is expressed as a single transcript containing the short form of WNT10B oncogene described here; a non-functional protein is predicted *in silico* however. For the first time these observations revealed the existence of a short version of the WNT10B oncogene, rarely integrated at the DNA level, which points to its potential involvement in the WNT10B^R^ generation by non-random microhomology-mediated recombination as recently suggested in virus-mediated integration^28^.

### Identification of the WNT10B-FZDs interacting complexes by *in situ* PLA in the MCF-7 cell line carrying the WNT10B^R^

WNT molecules can signal via binding to members of the seven-transmembrane-spanning Frizzled (FZD) receptors. Despite the involvement of aberrant Wnt signaling in a variety of human cancers, relatively little is known about the role of such receptors in cancer pathogenesis. Although we recently confirmed higher expression for FZD4 in AML^20^,^29^, it is still not known which FZD receptor(s) bind WNT10B. The MCF7 breast cancer cell line was selected for functional studies since it expresses the WNT10B^IVS1^ allele. By culturing them in conditions that form spheres they exhibit more cancer stem cell like features, e.g. expressing AC133^+^ that we in AML correlates to a cell population that responds to WNT10B via an autocrine/paracrine loop^8^.

**Figure 5:**
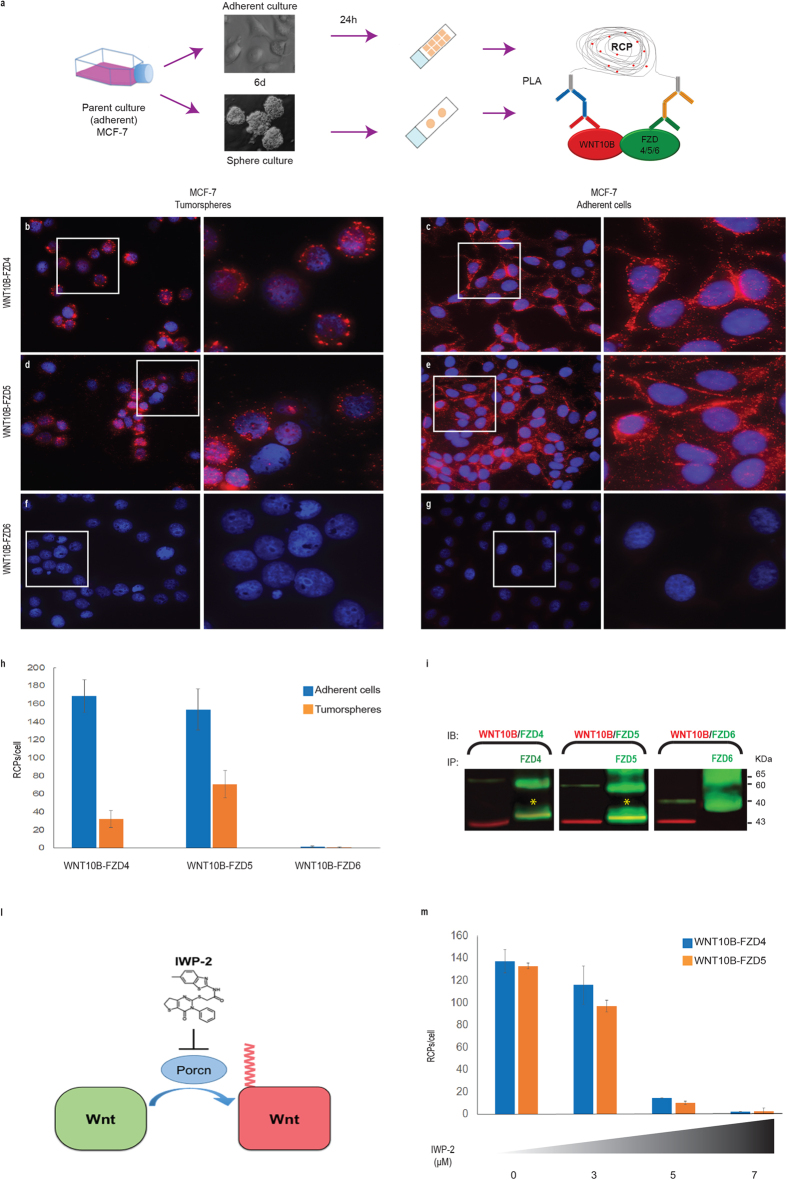
WNT10B-FZD4/5/6 complexes detection in MCF-7 cell line by *in situ* PLA. (**a**) Overview of the experimental design: adherent and tumorsphere culture of MCF-7 cell line were generated from subconfluent parent culture. In order to perform the *is*PLA analysis to the WNT10B-FZD4/5/6 complex detection, the tumorsphere cells were collected by cytospin while the adherent cells were fixed after six days on a slide. (**b**–**g**) Visualization of WNT10B-FZD4 (**b**,**c**), WNT10B-FZD5 (**d**,**e**) and WNT10B-FZD6 (**f**,**g**) interaction in MCF-7 tumorsphere (left) and adherent (right) cultured cells by *is*PLA. Formation of complexes are visualized using hybridization probes labeled with Alexa568 (red), and the nuclei are stained with Hoechst (blue). Images were acquired with Maximum-Intensity Projection (MIP) x40 magnification. Exposure times MCF-7 cells: DAPI 350 ms, Cy3 200 ms. Scale bars 10 μm. (**h**) The RCA signals were quantified using the Cell Profiler software, and data were plotted as a histogram which represents the RCPs per cell in MCF-7 adhesion cells (blue) and in MCF-7 tumorspheres (orange). Data represent mean values ± s.d. (**i**) Immunoblot (IB) analysis of MCF-7 cell lysate using α-WNT10B and α-FZD4, α-FZD5 and α-FZD6 antibodies (left side) and the WNT10B-FZD4, WNT10B-FZD5 and WNT10B-FZD6 protein complexes detection by Co-Immunoprecipitation assay (Co-IP) (right side). Co-Immunoprecipitation with α-FZD4, α-FZD5 and α-FZD6 antibodies, and immunoblotting with antibodies as indicated. The expected weight (KDa) of protein complexes are shown on the right side of panel. (**l**) IWP-2 chemical compound inactivates the *N*-palmitoyltransferase PORCN to selectively block the palmitoylation and secretion of the Wnt ligands. (**m**) MCF-7 cells were treated with IWP-2 chemical inhibitor (3 μM, 5 μM, 7 μM), thereafter complexes between WNT10B-FZD4 and WNT10B-FZD5 proteins were determined by the *in situ* PLA method. A significative concentration-dependent decrease in the number of RCPs per cell was detected in MCF-7 cells treated with IWP-2, as represented by data plotted in histogram. Data represent mean values ± s.d.

In order to visualize interactions between WNT10B and FZD4/5/6 we used proximity ligation assay (PLA) (Fig. 5a) in both adherent cultures and in tumorspheres which are significantly enriched for AC133 on MCF7. Compaction of the RCA products, resulted in high intensity signals, were detected for WNT10B-FZD4/5 complexes, both in tumorsphere (Fig. 5b,d,h) and in adherent (Fig. 5c,e,h) MCF7 cells (Extended data Fig. 6a,b,d,e). In contrast, *in situ* PLA analysis of WNT10B-FZD6 interaction, did not generate specific detection signals (Fig. 5f,g) in the MCF7 cell system (Supplementary Fig. 6c,f). Co-immunoprecipitation was performed to confirm the PLA results (Fig. 5i).

Finally, we used the IWP2 compound acting on the acyltransferase Porcupine^30^ (Fig. 5l) to selectively inhibit the Wnt autocrine loop since small molecule inhibitors of regulatory events underlying production of WNT proteins facilitate a chemical-based interrogation of pathway function. The level of *in situ* PLA signals for WNT10B-FZD4/5 was reduced in IWP2-treated cells in a dose-dependent manner (Fig. 5m).

These results not only point to FZD4/5 as WNT receptors specifically synergizing with WNT10B, but they also confirm an autocrine mechanism for Wnt signaling activation in a cellular model characterized by the presence of the rearranged WNT10B^R^ allele.

Overall, our data demonstrate the existence of a mobile element containing a ht-WNT10B short form, whose precise origin is unknown as are mechanisms involved in the transmission of this genetic element. These findings are consistent with recent evidence showing that exogenous DNA integrates in human somatic genomes more frequently in tumors than normal samples^31^. It is possible that viral and/or bacterial integrations by lateral gene transfer may lead to altered gene expression mainly in AML and breast cancers, as supported by recent data^32^. This would provide an additional avenue for viral/bacteria-associated oncogenesis, beyond inflammation-induced damage^33^.

## Methods

### Zebrafish

#### Fish and embryos maintenance

Embryos were handled according to relevant guidelines. Fish were maintained at 28 °C on a 14-hour light/10-hour dark cycle and collected by natural spawning.

We used embryos from the AB strain and *mpx*:GFP^34^ and *gata1*:DsRed^35^ transgenic lines. Our facility strictly complies with the relevant Italian laws, rules and regulations (Legislative Decree No. 116/92), as confirmed by the authorization issued by the municipality Milan (Art. 10 of Legislative Decree No. 116, dated 27.1.1992). All of the procedures were carried out in accordance with the relevant guidelines and regulations.

#### *wnt10b* cDNA cloning and transcription

Two specific primers (Zf_P1 and Zf_P2, See Extended Table 4) were used to amplify the *wnt10b* ORF using reverse transcribed total RNA extracted from zebrafish embryos and larvae at 24–96 hours post fertilization (hpf). The obtained cDNA fragment was gel purified and cloned into pCMV–SC (Stratagene, Agilent Technologies, Santa Clara, CA, USA). Capped mRNA was transcribed using the mMessage mMachine Kit (Ambion, Austin, TX-Thermo Fisher Scientific) according to the manufacturer’s instructions. All control embryos were obtained injecting the zygote with GFP or DsRed synthetic mRNA, depending on the fluorescence of the transgenic lines under analysis.

#### Microinjection and analyses

*wnt10b* transcript microinjections were carried out on 1- to 2-cell stage embryos using 400 pg of capped mRNA per embryo.

Whole mount *in situ* hybridization (WISH) was performed as previously described^36^ on embryos collected at the desired developmental stages and fixed in 4% paraformaldehyde/phosphate buffered saline, using *scl*^37^ and *pu*.*1*^38^ DIG-labeled riboprobes.

Live imaging of transgenic embryos was carried out using the Zeiss Axiovert 200 M. To determine the number of neutrophils in the *mpx*:GFP transgenics each embryo was photographed on 4–5 different focal planes, then the pictures were assembled in a single image on which the cell count was performed using the ImageJ 1.47 software.

### Patients and Sample Collection

We collected BM MNCs from n = 125 AML patients at diagnosis and according to the 2015 National Comprehensive Cancer Network (NCCN) classification, samples included n = 48 favorable risk, n = 70 intermediate-adverse risk and n = 7 Therapy-related (t-AML) AML patients. We collected also one prototypic case resulted affected by t-AML after a personal history of breast cancer followed and radiation therapy (XRT). Patients’ characteristics are shown in Extended Table 1 and Extended Table 2. Human BM cells obtained from n = 6 healthy donors were also collected. Each patient gave his/her informed consent for collection of clinical data, the cryopreservation of bone marrow samples and the performance of DNA-analysis for scientific purposes, in accordance with institutional guidelines. For each patient, data regarding history, haematologic parameters, bone marrow morphology, immunophenotype, cytogenetic, molecular analysis, and diagnosis of extra-medullary leukemia were recorded. Treatment schedule and outcome data were available for 116 out of 125 patients.

Besides, in this study, n = 2 non-familial breast cancer patients (NFBC) were recruited from Niguarda Hospital, Milan (Italy). Tumor and adjacent normal tissues of the same patient were obtained during resection and immediately snap-frozen in liquid nitrogen. Patients’ clinical and histopathological informations are summarized in Extended Table 3. The study design adhered to the Declaration of Helsinki and approval for this study was obtained from the Niguarda Hospital Review Board (116_04/2010).

### Definitions and criteria for treatment response

Complete remission (CR) was defined as less than 5% of bone marrow blasts, regression of extramedullary disease, transfusion independency with peripheral neutrophil count greater than 1000/μL and platelet count greater than 100000/μL and disappearance of the cytogenetic and molecular markers. Recurrent disease is defined as the reappearance of ≥5% blasts in the bone marrow or in the peripheral blood or as the appearance of a new extramedullary site of disease in patients with a previously documented CR. Extramedullary disease was defined as any leukemic collection outside the bone marrow and its presence was documented either by histological, cytological or radiological criteria.

Overall survival (OS) was calculated from the date of diagnosis until death, where all living patients were censored at the time of last contact. The duration of CR was calculated from the date of the first CR until the date of the first relapse. Relapse-free survival (RFS) was calculated from the date of the first CR until the date of the first relapse, where patients were censored at the time of last contact or death not due to recurrent disease.

### 5′ WNT10B Flanking Region analysis

#### Single Primer PCR

Genomic DNA was recovered using Dnazol (Invitrogen, Thermo Fisher), following manufacturer’s instructions. The reaction mixture of the Single Primer-PCR^39^ contained, in a total volume of 30 μl, 3 ng of DNA, 3 μl of 10X M-Buffer (500 mM Tris, 2.5 mg/ml BSA, 20 mM MgCl_2_, 5% Ficoll and 5 mM Cresol Red), 3 μl of 5 μM 1 P Primer and 3 μl of T/TS [10.5 ul of 2.5 mg/ml of BSA diluted in 100 mM Tris, 1 μl Taq Pol and 1 μl Hotstart Taq Pol, (NEB New England Biolabs, Mississauga, ON, Canada)]. The amplification was carried out in a Veriti thermal cycler (Applied Biosystems) as follows: 94 °C for 30 s, 20 cycles at 94° for 0 s, 55 °C for 0 s and 72 °C for 60 s; 30 cycles at 94° for 0 s, 55 °C for 0 s and 72 °C for 60 s; 30 cycles at 94° for 0 s, 55 °C for 0 s and 72 °C for 60 s. The PCR product was then analyzed by electrophoresis through a 1% agarose gel.

#### Sequence reaction

The purified templates were dissolved in sterile water and the Applied Biosystems Big-Dye ver. 1.1 chemistry kit was used to perform the sequencing reactions. Sequences were analysed on ABI3130XL automatic sequencers (Applied Biosystems, Foster City, CA, USA).

### WNT10B/WNT10B^IVS1^ Gene expression analysis

Total RNA was extracted using the RNAqueous-4PCR kit following the manufacturer’s instructions (Ambion, Austin, TX-Thermo Fisher Scientific), and then were reverse transcribed with RT mixtures containing 500 ng of RNA, 100 U Improm II (Promega, Madison, WI, USA), 1x buffer, 1 mM dNTPs, 5 mM MgCl_2_, and 0.5 μg of P5 reverse primer in a total volume of 20 μl for 5 min at 25 °C and then 60 min at 42 °C. The reaction was terminated by heating at 75 °C for 5 min. All PCRs were performed containing 5 μl of DNA as template, 5X Green GoTaq Buffer (Promega Corporation, Madison, WI, USA), 4 mM of MgCl_2_, 0.2 μM of each primer, 0.2 mM dNTPs (Promega), 0.5 U of Taq DNA polymerase (Promega), and water to a final volume of 50 μl. The GAPDH retrotranscription was performed using random primers. The WNT10B (P4-P2 primers) amplification was performed with following thermal conditions: 94 °C for 1 min, 33 cycles at 94° for 30 s, 58 °C for 30 s, 72 °C for 30 s and 72 °C for 5 min. The amplification of WNT10B^IVS1^ (P3-P2 primers) was performed as follows: 94 °C for 1 min, 33 cycles at 94° for 30 s, 61 °C for 30 s, 72 °C for 30 s and 72 °C for 5 min. The GAPDH (P6-P7 primers) housekeeping gene amplification was carried out in a thermal cycler as follows: 94 °C for 1 min, 30 cycles at 94° for 20 s, 60 °C for 15 s, 72 °C for 15 s and 72 °C for 5 min. Primers are listed in Extended Table 4. At the end the PCR products were analysed by electrophoresis through a 2% agarose gel.

#### WNT10B/WNT10B^IVS1^ Absolute quantification

In our study, ddPCR^40^ experiments were performed in blind way at the BIORAD laboratories (Milan, Italy) using primers and probes listed in Extended Table 4. The 0.1 mM RNA, extracted using the RNAqueous-4PCR kit following the manufacturer’s instructions (Ambion, Austin, TX-Thermo Fisher Scientific), was denatured at 95 °C for 5 min and kept on ice prior addition to the reaction. We performed the experiment on Bio-Rad’s QX100 ddPCR system and the reaction mixtures in a final 20 μl volume consisted of 10 μl of 2× One-Step RT-ddPCR Supermix (Bio-Rad, CA, USA), 1 mM Manganese Acetate solution (Bio-Rad, CA, USA), 0.5 μM of primers (WNT10B: P4-P2, WNT10B^IVS1^ P3-P2), 0.25 μM WNT10B_dd1 and WNT10B^IVS1^_dd2 probes. The 20 μL ddPCR reaction mixture was then loaded into the Bio-Rad DG8 droplet generator cartridge (Bio-Rad, CA, USA). Then, each oil well was filled with 70 μl of droplet generation oil (Bio-Rad, CA, USA) and the prepared cartridge was then loaded into the QX100 droplet generator (Bio-Rad, CA, USA). The generated droplets were transferred to a 96-well PCR plate, and then samples were amplified on the T100 BioRad thermal cycler. The thermal cycling conditions consisted of 30 min reverse transcription at 60 °C, 5 min initial denaturation at 95 °C, followed by 40 cycles of a two-step thermal profile of 30 s denaturation at 94 °C and 60 s annealing-elongation at 60 °C and a final 10 min denaturation step at 98 °C. Then plates were transferred to the QX 100 droplet reader (Bio-Rad, CA, USA) and ddPCR data were analyzed with QuantaSoft analysis software (version 1.7.4).

### Statistical analysis

All collected variables were submitted to usual descriptive methods. In particular, for continuous variables the distribution was firstly evaluated by the Shapiro-Wilk test, so that normally distributed variables were summarized with mean and standard deviation, while non-normal variables were summarized with median and range. The Pearson’s chi-square test with Yates’ correction for continuity and the Fisher’s exact test were used to check the association between categorical data, after cross-tabulation. Comparisons of normally distributed continuous variables were carried out by Student’s t-test or by Welch test (in the case of non-homogeneous variances between groups, previously verified by Levene’s test). The Kruskal-Wallis test and the Mann-Whitney U-test were used for comparison of continuous non-normally distributed variables.

The survival analysis was carried out using the Kaplan-Meier product limit method, followed by the logrank test, to evaluate the possible differences in survival between groups. Cox univariate and multivariate regression models were also used to analyse the effects of continuous variables on survivorship. The optimal multivariate model was chosen using a backward stepwise elimination after inserting all variables showing p < 0.20 at univariate analysis.

The receiver operating characteristics curve (ROC) was traced to analyse the role of WNT transcript levels on survivorship and to search for an optimal cut-off value for WNT transcript itself. For all possible cut-off points, the total accuracy was considered together with sensitivity, specificity, positive predictive value and negative predictive value; however, the choice was made according to Youden.

Statistical analysis was done using MedCal 9.3.7.0 and statistical significance was assumed for all tests with p < 0.05.

Patients subgroups were identified in gene expression data using consensus clustering, named “clustercons”, by R-package. Corresponding scatterplots were produced using the scatterplot3d (https://cran.r-project.org/web/packages/scatterplot3d/index.html).

### mRNA *in situ* detection

All mRNA *in situ* experiments were performed according to *Larsson et al.*^41^ with modifications, in ordinance with *guidelines* established in the related patent (US 8551708 B2). Informed consents for *in situ* expression analysis on bone marrow biopsies were obtained, this study was approved by the Niguarda Hospital Review Board (116_04/2010).

Bone marrow biopsies of AML patients, previously embedded in paraffin blocks, were cut in 5 μm thick sections and mounted on slides. Slides were dewaxed as follows: twice in 100% xylene for 15 minutes and 10 minutes, twice in 100% EtOH for 2 minutes, twice in 95% EtOH for 2 minutes, twice in 70% EtOH for 2 minutes, and washed in DEPC-H_2_O for 5 minutes and in DEPC-PBS for 2 minutes. Tissue fixation was performed in 3.7% (w/v) paraformaldehyde in PBS for 10 minutes at room temperature. After a wash in DEPC‐PBS for 2 minutes, the tissue sections were then permeabilized with 2 mg/ml pepsin (Sigma Aldrich, St. Louis, US) in 0.1 M HCl at 37 °C for 2 minutes. Slides were washed in DEPC-H_2_O for 5 minutes, in DEPC-PBS for 2 minutes and then fixed in 3.7% (w/v) paraformaldehyde in PBS for 10 minutes at room temperature. Tissue sections were then dehydrated through a series of 70%, 85% and 100% ethanol for 1 min each. The *in situ* reactions were carried out with a reaction volume of 100 μl in secure-seals (13 mm in diameter and 0.8 mm deep; Grace Bio-Labs) mounted over the tissue. One μM of locked nucleic acid (LNA)-modified cDNA primer (Exiqon, Vedbaek, Denmark; P8 and P9, see Extended Table 4) was added to the slide with 10 U/μl of M‐MULV reverse transcriptase (Fermentas), 500 nM dNTPs (Invitrogen), 0.2 μg/μl BSA (New England Biolabs, NEB) and 1 U/μl RiboLock RNase Inhibitor (Fermentas) in the 1X M-MULV reaction buffer. Slides were incubated for 3 hours at 37 °C. After incubation, slides were washed in PBS-T (DEPC‐PBS with 0.05% Tween20), followed by a post-fixation step in 3.7% (w/v) paraformaldehyde in DEPC‐PBS for 30 min at room temperature. Then the samples were washed twice in DEPC PBS-T. Ligation was then carried out with 0.1 μM of the β‐actin, WNT10B and WNT10B^IVS1^ padlock probes (Sigma-Aldrich, St Louis, MO; PP1, PP2 and PP3, see Extended Table 4) and in a mix of 0.5 U/μl Ampligase (Epicentre), 0.4 U/μl RNase H (Fermentas), 1 U/μl RiboLock RNase Inhibitor (Fermentas), Ampligase buffer, 50 mM KCl and 20% formamide. Incubation was performed first at 37 °C for 30 minutes, followed by 45 minutes at 45 °C. After ligation reaction, the slides were washed twice in DEPC-PBS with 0.05% Tween20 and then the Rolling Circle Amplification (RCA) was performed with 1 U/μl DNA Polymerase (Fermentas) in the supplied reaction buffer, 1 U/μl RNase Inhibitor (Fermentas), 250 μM dNTPS (Invitrogen), 0.2 μg/μl BSA (NEB) and 5% glycerol. Incubation was carried out for 7 hours at 37 °C, and it was followed by a twice wash in PBS-T. Rolling Circle Particles (RCPs) were visualized using 100 nM of detection probe (Sigma-Aldrich; DP1 and DP2, see Extended Table 4) in 2X SSC and 20% formamide at 37 °C for 20 minutes. Slides were then washed in DEPC-PBS. Nuclei were counterstained with 100 ng/ml Hoechst 33258 (Sigma‐Aldrich). The Secure-seals were removed and the slides were dehydrated using a series of 70%, 85% and 100% ethanol for 3 minutes each. The dry slides were mounted with Invitrogen Slowfade. Images of bone marrow tissue slides were acquired using an Axioplan II epifluorescence microscope (Zeiss) equipped with a 100 W mercury lamp, a CCD camera (HRM, Zeiss), and a computer-controlled filter wheel with excitation and emission filters for visualization of DAPI, Cy3, and Cy5 at room temperature of 25 °C. A x20 (Plan Apocromat, Zeiss) and x40 (Plan Neofluar, Zeiss) objective were used for capturing the images. Images were collected using the Axiovision software (release 4.3, Zeiss). Exposure times for slides images were 520–680 ms (at 20X magnification), 320–480 ms for DAPI; 300–650 ms for Cy3; 580 ms for Cy5. Images were collected as z-stacks to ensure that all RCPs were acquired, with a maximum intensity project (MIP) created in Axiovision. For quantification, the numbers of RCPs and cell nuclei in three different images were counted digitally using CellProfiler (www.cellprofiler.org) on three x20 microscope images. The average for each sample was then calculated from the result of the three images and is reported as RCPs per cell. The threshold for different color channels was set with ImageJ 1.41.

### Molecular characterization of WNT10B^R^ and htWNT10B

All WNT10B^R^ and ht-WNT10B genomic amplifications were performed using the reaction mixture of 50 μl consisted in 500 ng of DNA, 1× GoTaq Buffer, 1.5 mM MgCl_2_, 0.2 μM of P2, P1 and P5 primers listed in Extedended Table 4, 0.5 mM of dNTPs and 1U of TaqDNA Polymerase (Promega, Madison, WI, USA). Total RNA was extracted and reverse transcribed using the protocol previous described and PCRs were performed in a final volume of 50 μl containing 5 μl of DNA as template, 5X Green GoTaq Buffer (Promega Corporation, Madison, WI, USA), 4 mM of MgCl_2_, 0.2 μM of each primer, 0.2 mM dNTPs (Promega), 0.5 U of Taq DNA polymerase (5 U/μl; Promega). The amplifications were performed with following thermal conditions: P2-P1, 94 °C for 3 min, 30 cycles at 94° for 30 s, 58 °C for 30 s, 72 °C for 30 s and 72 °C for 5 min; P2-P10, 94 °C for 5 min, 33 cycles at 94° for 30 s, 59 °C for 45 s, 72 °C for 5 min and 72 °C for 10 min. Then the PCR products were visualized by 0.8% and 1.5% electrophoresis agarose gels and sequenced using the chemistry described above. The obtained P2-P10 fragment was gel purified and cloned into TOPO® TA Cloning® Kit (Invitrogen, Carlsbad, CA). We amplified GAPDH as housekeeping gene, following the protocol described above.

### MCF-7 Adherent and Tumorspheres cell culture

The human breast adenocarcinoma cells (MCF-7) were purchased from ATCC (Manassas, VA) and was authenticated by STR profiling, using the AmpFlSTR® Identifiler kit and tested for mycoplasma (MycoProbe from RnD # number CUL001B). We grew MCF-7 cells in T-25 flasks (Corning) in DMEM (Gibco), supplemented with 10% (v/v) fetal bovine serum (FBS), 10 μg/ml insulin, 2 mM L-Glutamine and 0.02 μg/ml epidermal growth factor (EGF) (Sigma-Aldrich, USA). For slide preparation we trypsinized cells and seeded them into eight-well chamber slides (Lab-Tek, Nunc), allowing theme to adhere for 48 h. At the second day, medium was then aspirated and cells were fixed in 3.7% formaldehyde solution for 30 min on ice. Then, after a step to was away residual of fixative solution, we dried slides in absolute ethanol and stored them at −20 °C until further use. In order to generate tumorspheres, adherent cells were trypsinized with 0.05% Trypsin-EDTA (Gibco), washed three times with calcium-magnesium-free PBS and after adding 2 mL of DMEM/F12, tumorspheres were dissociated by gentle pipetting^42^. Cells are then seeded at concentration of 5 × 10^4 ^cells/ml in low adherent T-25 flasks (Corning, Manassas, VA), in NSA media [DMEM/F12, (Gibco), 20 ng/mL EGF (Sigma), 10 ng/mL recombinant human basic fibroblast growth factor bFGF (R&D Systems), 4 μg/ml heparin (Sigma), 10% human NeuroCult® (Stem Cell Technologies)], 0.15% bovine serum albumin BSA (Sigma) and 1% penicillin G-streptomycin solution (Gibco). Cells were grown at 37 °C in a humidified atmosphere with 5% CO_2_, for 6 days.

### Inhibition of WNT palmitoyltransferase

MCF-7 cells were plated at a concentration of 1 × 10^3 ^cells/well on Lab-Tek II (Nunc) chamber slides. Cells were serum starved for 48 h and then the Wnt palmitoyltransferase inhibitor (IWP-2), (Sigma Aldrich, St. Louis, MO, USA) was used at 0 μM, 3 μM, 5 μM, 7 μM concentrations to treat cells, in a dose dependent manner.

### *In situ* Proximity Ligation Assay

*In situ* PLA was performed according to the manufacturer’s instructions (Olink Bioscience), briefly the cells were permeabilized with 0.5% Triton X-100 for 10 min at room temperature (RT) and washed two times for 5 min in PBS. Slides were incubated with blocking solutions for 1 h at 37 °C and then were incubated with the primary antibodies (mouse monoclonal α-WNT10B [5 A7], Abcam; rabbit polyclonal α-FZD4, Abcam; rabbit polyclonal α-FZD5, Abcam; rabbit polyclonal α-FZD6 ThermoFisher Scientific) diluted in Duolink Blocking Solution (Olink Bioscience) for 2 h at room temperature. After overnight incubation at 4 °C with the antibodies, the samples were washed three times for 5 min in TBS plus 0.05% Tween 20 and therefore Duolink secondary probes Anti-Rabbit (Minus) and Anti-Mouse (Plus) probes (Olink Bioscience) were diluted (1:5) in Duolink Antibody Diluent (Olink Bioscience) and incubated over slides for 1 h at 37 °C. Then the slides were washed three times in TBS plus 0.05% Tween 20 for 10 min. Duolink ligase was added in Duolink Ligation Mix diluted to 1× in high purity water and then incubated for 30 min at 37 °C. Then samples were washed gently two times for 2 min in TBS plus 0.05% Tween 20 before the following amplification step. We performed the RCA with 25 nM of the compaction oligonucleotide^43^ (oligonucleotides C1 and D1, See Extended Table 4). The RCA took place in the presence of 25 nM of detection oligonucleotides in a RCA buffer containing 0.25 mg/ml BSA, 7.5 ng/ml poly-adenosine, 1× phi29 DNA polymerase buffer (Fermentas), 0.25 mM dNTPs (Thermo Scientific) and 0.25 U/μl phi29 DNA polymerase, followed by incubation at 37 °C for 1 h. In order to stain nuclei, we used 100 ng/ml of Hoechst (Sigma-Aldrich) and samples were incubated for 10 min at RT and then washed two times for 5 min each with TBS plus 0.05% Tween 20 and finally 5 min with TBS. After washing we dehydrated the slide by washing with an EtOH solution (70% EtOH and 30% TBS) for 2 min and secondly with a 99% solution of EtOH for 2 min. Slides were mounted with SlowFade Gold mounting medium (Invitrogen). Technical controls of *is*PLA, based on the omission of first antibodies in order to exclude possible cross-reactivity of secondary antibodies or non-specific PLA-signal generation, were performed (data not shown). The images were acquired as *z*-stacks with Zeiss AxioPlan2 microscope with a 20×/0.8 Plan-Apo objective and an Axiocam MRm camera and processed with Zeiss AxioVision 4.8 software at room temperature of 25 °C. In order to quantify RCPs/cells we used the CellProfiler software (www.cellprofiler.org) on five different x20 microscope images acquired.

### Co-Immunoprecipitation

Cells cultured in T-25 flasks, were washed with 0.5 ml ice-cold Cell Lysis Buffer adding 1 mM PMSF (Cell Signaling, Danvers, MA, USA)] with PhosSTOP (Roche), for 5 minutes. Then cells were gently scraped and collected in Eppendorf tubes and finally centrifuged for 10 min at 14,000 *g*, 4 °C. In order to measure the protein concentration in supernatant cell lysate, the Pierce™ BCA Protein Assay Kit (Pierce Biotechnology, Rockford, IL, USA) was used. Then, 100 μg of protein were incubated under rotation for 2 h at 4 °C with an antibody dilution of 1:100 (mouse monoclonal α-WNT10B [5 A7], Abcam; rabbit polyclonal α-FZD4, Abcam; rabbit polyclonal α-FZD5, Abcam; rabbit polyclonal α-FZD6 ThermoFisher Scientific). A total volume of 20 μl of 50% bead slurry Protein G Sepharose 4 Fast Flow beads (VWR, Wayne, NJ, USA) were added and incubated under rotation for 2 h at 4 °C. After three washing times with 500 μL cell lysis buffer the pellet was resuspended in 20 μl of SDS sample buffer (Cell Signaling), vortexed and centrifuged for 30 s, and finally incubated for 5 min at 95 °C. Samples were loaded on a NuPAGE 10% Bis-Tris Gel (Life Technologies) and gels were run at 130 V. In order to transfer proteins from the NuPAGE Gel to nitrocellulose membrane, an iBlot dry blotting system (Life Technologies) and iBlot Gel Transfer Stacks Nitrocellulose (Life Technologies) were used following the manufacturer’s instructions. Then the nitrocellulose membranes were blocked in LI-COR blocking buffer (LI-COR, Lincoln, NE, USA) for 1 h in RT, and primary antibodies were diluted 1:1000 in LI-COR blocking buffer and finally incubated with the membrane under gentle rotation overnight at 4 °C. After that the membrane was washed three times in 1× TBS supplemented with 0.05% Tween 20, secondary antibodies IRDye 680LT Donkey (polyclonal) Anti-Mouse IgG (H + L) Highly Cross Adsorbed (LI-COR) and IRDye 800CW Conjugated Goat (polyclonal) Anti-Rabbit (H + L) Highly Cross Adsorbed (LI-COR) were diluted 1:20,000 in LI-COR blocking buffer and incubated with the membrane under gentle rotation for 10 min at RT, followed by three washes in 1 × TBS supplemented with 0.05% Tween. Therefore we scanned membranes in the Odyssey infrared imaging system (LI-COR).

### Frizzled receptors gene expression analysis

Total RNA was extracted and reverse transcribed using the protocol previous described and random primers. All PCRs were performed in a final volume of 50 μl containing 5 μL of DNA as template, 5X Green GoTaq Buffer (Promega Corporation, Madison, WI, USA), 4 mM of MgCl_2_, 0.2 μM of each primer (See Extended Table 4), 0.2 mM dNTPs (Promega), 0.5 U of Taq DNA polymerase (5 U/μl; Promega Corporation). The frizzled receptors gene expression analysis was performed using the following thermal cycle conditions: P12-P13, 94 °C for 3 min, 33 cycles at 94° for 30 s, 52 °C for 30 s, 72 °C for 30 s and 72 °C for 2 min; P14-P15, 94 °C for 3 min, 33 cycles at 94° for 30 s, 52 °C for 30 s, 72 °C for 30 s and 72 °C for 2 min; P16-P17, 94 °C for 3 min, 33 cycles at 94° for 30 s, 51 °C for 30 s, 72 °C for 30 s and 72 °C for 2 min; P18-P19, 94 °C for 3 min, 33 cycles at 94° for 30 s, 53 °C for 30 s, 72 °C for 30 s and 72 °C for 2 min. The GAPDH amplification was performed as standard control. The PCR products were visualized by electrophoresis through a 2% agarose gel.

## Additional Information

**How to cite this article**: Lazzaroni, F. *et al.* Intronless WNT10B-short variant underlies new recurrent allele-specific rearrangement in acute myeloid leukaemia. *Sci. Rep.* **6**, 37201; doi: 10.1038/srep37201 (2016).

**Publisher’s note:** Springer Nature remains neutral with regard to jurisdictional claims in published maps and institutional affiliations.

## Supplementary Material

Supplementary Information

## References

[b1] Greaves M. & Maley C. C. Clonal evolution in cancer. Nature 481, 306–313 (2012). 22258609 10.1038/nature10762 PMC3367003

[b2] Congdon K. L. *et al.* Activation of Wnt signaling in hematopoietic regeneration. Stem Cells 26, 1202–1210 (2008). 18308947 10.1634/stemcells.2007-0768

[b3] Clevers H., Loh K. M. & Nusse R. An integral program for tissue renewal and regeneration: Wnt signaling and stem cell control. Science 346, 1248012 (2014). 25278615 10.1126/science.1248012

[b4] Reya T. *et al.* A role for Wnt signaling in self-renewal of haematopoietic stem cells. Nature 423, 409–414 (2003). 12717450 10.1038/nature01593

[b5] Luis T. C. *et al.* Canonical Wnt signaling regulates hematopoiesis in a dosage-dependent fashion. Cell Stem Cell 9, 346–356 (2011). 10.1016/j.stem.2011.07.017 21982234

[b6] Schreck C., Bock F., Grziwok S., Oostendorp R. A. J. & Istvanffy R. Regulation of hematopoiesis by activators and inhibitors of Wnt signaling from the niche. Ann N Y Acad Sci 1310, 32–43 (2014). 24611828 10.1111/nyas.12384

[b7] Zeng Y. A. & Nusse R. Wnt proteins are self-renewal factors for mammary stem cells and promote their long-term expansion in culture. Cell Stem Cell 6, 568–577 (2010). 20569694 10.1016/j.stem.2010.03.020 PMC2917779

[b8] Khramtsov A. I. *et al.* Wnt/b-catenin pathway activation is enriched in basal-like breast cancers and predicts poor outcome. Am. J. Pathol. 176, 2911–2920 (2010). 20395444 10.2353/ajpath.2010.091125 PMC2877852

[b9] Bafico A. *et al.* An autocrine mechanism for constitutive Wnt pathway activation in human cancer cells. Cancer Cell 6, 497–506 (2004). 15542433 10.1016/j.ccr.2004.09.032

[b10] Wang Y. *et al.* The Wnt/b-catenin pathway is required for the development of leukemia stem cells in AML. Science 327, 1650–1653 (2010). 20339075 10.1126/science.1186624 PMC3084586

[b11] Mikesch J. H., Steffen B., Berdel W. E., Serve H. & Muller-Tidow C. The emerging role of Wnt signaling in the pathogenesis of acute myeloid leukemia. Leukemia 21, 1638–1647 (2007). 17554387 10.1038/sj.leu.2404732

[b12] Eaves C. J. & Humphries R. K. Acute myeloid leukemia and the Wnt pathway. N. Engl. J. Med. 362, 2326–2327 (2010). 20554989 10.1056/NEJMcibr1003522

[b13] Lane S. W. *et al.* Differential niche and Wnt requirements during acute myeloid leukemia progression. Blood 118, 2849–2856 (2011). 21765021 10.1182/blood-2011-03-345165 PMC3172801

[b14] Luis T. C., Ichii M., Brugman M. H., Kincade P. & Staal F. J. T. Wnt signaling strength regulates normal hematopoiesis and its deregulation is involved in leukemia development. Leukemia 26, 414–421 (2012). 22173215 10.1038/leu.2011.387 PMC3378318

[b15] Lento W., Congdon K., Voermans C., Kritzik M. & Reya T. Wnt signaling in normal and malignant hematopoiesis. Cold Spring Harb Perspect Biol 5(2), 1–10 (2013). 10.1101/cshperspect.a008011 PMC3552513 23378582

[b16] Miharada K. & Karlsson S. Common signaling networks characterize leukemia-initiating cells in acute myeloid leukemia. Cell Stem Cell 10, 109–110 (2012). 22305558 10.1016/j.stem.2012.01.008

[b17] Beachy P. A., Karhadkar S. S. & Berman D. M. Tissue repair and stem cell renewal in carcinogenesis. Nature 432, 324–331 (2004). 15549094 10.1038/nature03100

[b18] Wend P., Wend K., Krum S. A. & Miranda-Carboni G. A. The role of WNT10B in physiology and disease. Acta Physiologica 204, 34–51 (2012). 21447090 10.1111/j.1748-1716.2011.02296.x

[b19] Wend P. *et al.* WNT10B/β-catenin signaling induces HMGA2 and proliferation in metastatic triple-negative breast cancer. EMBO Mol. Med. 5, 264–279 (2013). 23307470 10.1002/emmm.201201320 PMC3569642

[b20] Beghini A. *et al.* Regeneration-associated WNT signaling is activated in long-term reconstituting AC133^bright^ acute myeloid leukemia cells. Neoplasia 14, 1236–1248 (2012). 23308055 10.1593/neo.121480 PMC3540948

[b21] van de Water S. *et al.* Ectopic Wnt signal determines the eyeless phenotype of zebrafish masterblind mutant. Development 128, 3877–3888 (2001). 11641213 10.1242/dev.128.20.3877

[b22] Chen A. T. & Zon L. I. Zebrafish blood stem cells. J. Cell. Biochem. 108, 35–42 (2009). 19565566 10.1002/jcb.22251

[b23] Jan M. *et al.* Clonal evolution of preleukemic hematopoietic stem cells precedes human acute myeloid leukemia. Sci. Transl. Med. 4, 149ra118 (2012). 10.1126/scitranslmed.3004315 PMC4045621 22932223

[b24] Nardi V. *et al.* Acute myeloid leukemia and mielodysplastic syndromes after radiation therapy are similar to de novo disease and differ from other therapy-related myeloid neoplasms. J. Clin. Oncol. 30, 2340–2347 (2012). 22585703 10.1200/JCO.2011.38.7340 PMC4979234

[b25] Lane T. F. & Leder P. *Wnt-10b* directs hypermorphic development and transformation in mammary glands of male and female mice. Oncogene 15, 2133–2144 (1997). 9393971 10.1038/sj.onc.1201593

[b26] Huarte M. The emerging role of lncRNA in cancer. Nat. Med. 21, 1253–1261 (2015). 26540387 10.1038/nm.3981

[b27] Garzon R. *et al.* Expression and prognostic impact of lncRNA in acute myeloid leukemia. Proc. Natl. Acad. Sci. USA 111, 18679–18684 (2014). 25512507 10.1073/pnas.1422050112 PMC4284555

[b28] Hu Z. *et al.* Genome-wide profiling of HPV integration in cervical cancer identifies clustered genomic hot spots and a potential microhomology-mediated integration mechanism. Nat. Genet. 47, 158–163. 25581428 10.1038/ng.3178

[b29] Tickenbrock L. *et al.* Activation of Wnt signaling in acute myeloid leukemia by induction of Frizzled-4. Int. J. Onc. 33, 1215–1221 (2008). 19020754

[b30] Chen B. *et al.* Small molecule-mediated disruption of Wnt-dependent signaling in tissue regeneration and cancer. Nat. Chem. Biol. 5, 100–107 (2009). 19125156 10.1038/nchembio.137 PMC2628455

[b31] Riley D. R. *et al.* Bacteria-human somatic cell lateral gene transfer is enriched in cancer samples. PLoS Comput. Biol. 9, e1003107 (2013). 23840181 10.1371/journal.pcbi.1003107 PMC3688693

[b32] Robinson K. M. *et al.* A review of bacteria-animal lateral gene transfer may inform our understanding of diseases like cancer. PLoS Genet. 9, e1003877 (2013). 24146634 10.1371/journal.pgen.1003877 PMC3798261

[b33] Robinson K. M. & Dunning Hotopp J. C. Mobile elements and viral integrations prompt considerations for bacterial DNA integration as a novel carcinogen. Cancer Lett. 352, 137–144 (2014). 24956175 10.1016/j.canlet.2014.05.021 PMC4134975

[b34] Renshaw S. A. *et al.* A transgenic zebrafish model of neutrophilic inflammation. Blood 108, 3976–3978 (2006). 16926288 10.1182/blood-2006-05-024075

[b35] Traver D. *et al.* Transplantation and *in vivo* imaging of multilineage engraftment in zebrafish bloodless mutants. Nat. Immunol. 4, 1238–1246 (2003). 14608381 10.1038/ni1007

[b36] Thisse C. *et al.* Structure of the zebrafish snail1 gene and its expression in wild type, spadetail and no tail mutant embryos. Development 119, 1203–1215 (1993). 8306883 10.1242/dev.119.4.1203

[b37] Gering M., Rodaway A. R. F., Gottgens B., Patient R. K. & Green A. R. The SCL gene specifies haemangioblast development from early mesoderm. EMBO J. 17, 4029–4045 (1998). 9670018 10.1093/emboj/17.14.4029 PMC1170736

[b38] Lieschke G. J. *et al.* Zebrafish SPI-1 (PU.1) marks a site of myeloid development independent of primitive erythropoiesis: implications for axial patterning. Dev.Biol. 246, 274–295 (2002). 12051816 10.1006/dbio.2002.0657

[b39] Hermann S. R. J. A. M. *et al.* Single primer amplification of flanking sequences. Biotechniques 29, 1176–1180 (2000). 11126116 10.2144/00296bm04

[b40] Pinheiro L. B. *et al.* Evaluation of a Droplet Digital Polymerase Chain Reaction Format for DNA Copy Number Quantification. Analytical Chemistry 84(2), 1003–1011 (2012). 22122760 10.1021/ac202578x PMC3260738

[b41] Larsson C. *et al.* *In situ* detection and genotyping of individual mRNA molecules. Nat Methods 7, 395–397 (2010). 20383134 10.1038/nmeth.1448

[b42] Smart C. E. *et al.* *In vitro* analysis of breast cancer cell line tumourspheres and primary human breast epitelia mammospheres demonstrates inter- and intrasphere heterogeneity. Plos One 8(6), e64388 (2013). 23750209 10.1371/journal.pone.0064388 PMC3672101

[b43] Clausson C.-M. *et al.* Compaction of rolling circle amplification products increases signal integrity and signal-to-noise ratio. Sci. Rep. 5, 12317 (2015). 26202090 10.1038/srep12317 PMC4511876

