# Corrigendum: Intronless WNT10B-short variant underlies new recurrent allele-specific rearrangement in acute myeloid leukaemia

**DOI:** 10.1038/srep46788

**Published:** 2017-04-26

**Authors:** Francesca Lazzaroni, Luca Del Giacco, Daniele Biasci, Mauro Turrini, Laura Prosperi, Roberto Brusamolino, Roberto Cairoli, Alessandro Beghini

Scientific Reports 6: Article number: 3720110.1038/srep37201; published online: 09 17 2016; updated: 04 24 2017

This Article contains typographical errors in the Methods section under subheading ‘**WNT10B/WNT10B**^**IVS1**^ **Gene expression analysis’**.

“The WNT10B (P4-P2 primers) amplification was performed with following thermal conditions: 94 °C for 1 min, 33 cycles at 94° for 30 s, 58 °C for 30 s, 72 °C for 30 s and 72 °C for 5 min. The amplification of WNT10BIVS1 (P3-P2 primers) was performed as follows: 94 °C for 1 min, 33 cycles at 94° for 30 s, 61 °C for 30 s, 72 °C for 30 s and 72 °C for 5 min”.

should read:

“The WNT10B (P4-P1 primers) amplification was performed with following thermal conditions: 94 °C for 1 min, 33 cycles at 94° for 30 s, 58 °C for 30 s, 72 °C for 30 s and 72 °C for 5 min. The amplification of WNT10BIVS1 (P3-P1 primers) was performed as follows: 94 °C for 1 min, 33 cycles at 94° for 30 s, 61 °C for 30 s, 72 °C for 30 s and 72 °C for 5 min”.

In the same section, under subheading ‘**WNT10B/WNT10B**^**IVS1**^ **Absolute quantification**’,

“We performed the experiment on Bio-Rad’s QX100 ddPCR system and the reaction mixtures in a final 20 μl volume consisted of 10 μl of 2 × One-Step RT-ddPCR Supermix (Bio-Rad, CA, USA), 1 mM Manganese Acetate solution (Bio-Rad, CA, USA), 0.5 μM of primers (WNT10B: P4-P2, WNT10B^IVS1^ P3-P2), 0.25 μM WNT10B_dd1 and WNT10B^IVS1^_dd2 probes”.

should read:

“We performed the experiment on Bio-Rad’s QX100 ddPCR system and the reaction mixtures in a final 20 μl volume consisted of 10 μl of 2 × One-Step RT-ddPCR Supermix (Bio-Rad, CA, USA), 1 mM Manganese Acetate solution (Bio-Rad, CA, USA), 0.5 μM of primers (WNT10B: P4-P1, WNT10B^IVS1^ P3-P1), 0.25 μM WNT10B_dd1 and WNT10B^IVS1^_dd2 probes”.

