# Supplementary material for: Intronless WNT10B-short variant underlies new recurrent allele-specific rearrangement in acute myeloid leukaemia
Source: Sci Rep. 2016 Nov 17;6:37201. doi: 10.1038/srep37201 (PMC5112549; doi:10.1038/srep37201)
Supplement: Supplementary Information [file srep37201-s1.pdf]

Intronless WNT10B-short variant underlies new recurrent allele-specific rearrangement in acute myeloid leukaemia

Francesca Lazzaroni[1], Luca Del Giacco[2], Daniele Biasci[3], Mauro Turrini[4], Laura Prosperi[2], Roberto Brusamolino[5], Roberto Cairoli[6], Alessandro Beghini[1]

[1]Department of Health Sciences, University of Milan, Milan, Italy.
[2]Department of Biosciences, University of Milan, Milan, Italy.
[3]Cambridge Institute for Medical Research, University of Cambridge, Cambridge, UK
[4]Department of Internal Medicine, Valduce Hospital, Como, Italy
[5]Department of Pathology, Niguarda Hospital, Milan, Italy.
[6]Department of Oncology, Hematology Unit, Niguarda Hospital, Milan, Italy.

Corresponding Author:
Alessandro Beghini
University of Milan, Medical Faculty
Department of Health Sciences
Via Rudinì, 8
Milan 20142, Italy
Tel +39 02 503.23230; email: alessandro.beghini@unimi.it

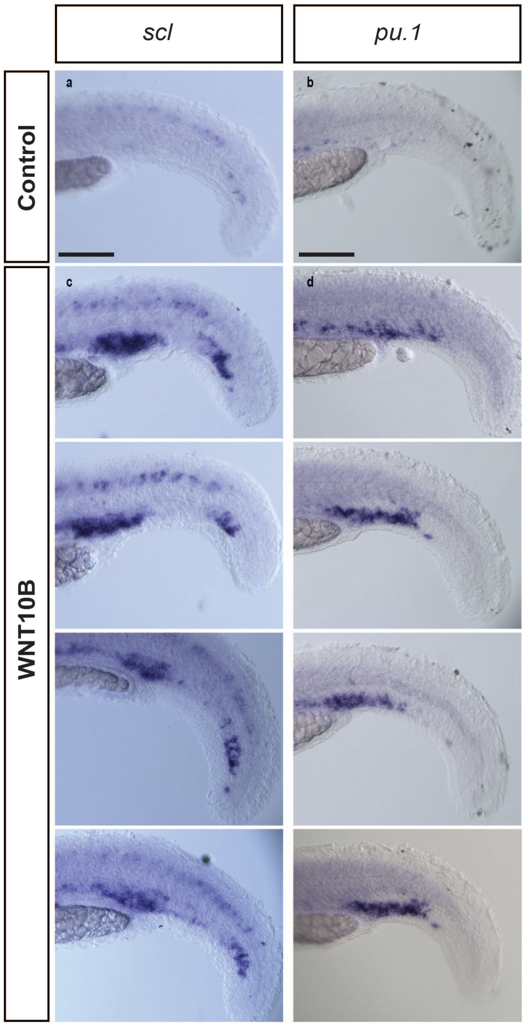

Supplementary Figure 1. *wnt10b* overexpression promotes the accumulation of the hematopoietic precursors in the PBI.

a, b, Magnification of representative whole mount *in-situ* hybridizations to *scl* and *pu.1* on 28 hours post fertilization (hpf) **c**, **d**, *wnt10b*-injected and control embryos. *Wnt10b*-overexpressing embryos display a higher number of *scl*- and *pu.1*-expressing cells in the PBI (see also Figure 1). Some of the images are composed of different pictures corresponding to several focal planes, since a single focal plane cannot comprise all the labeled areas. Scale bar, 50 μm.

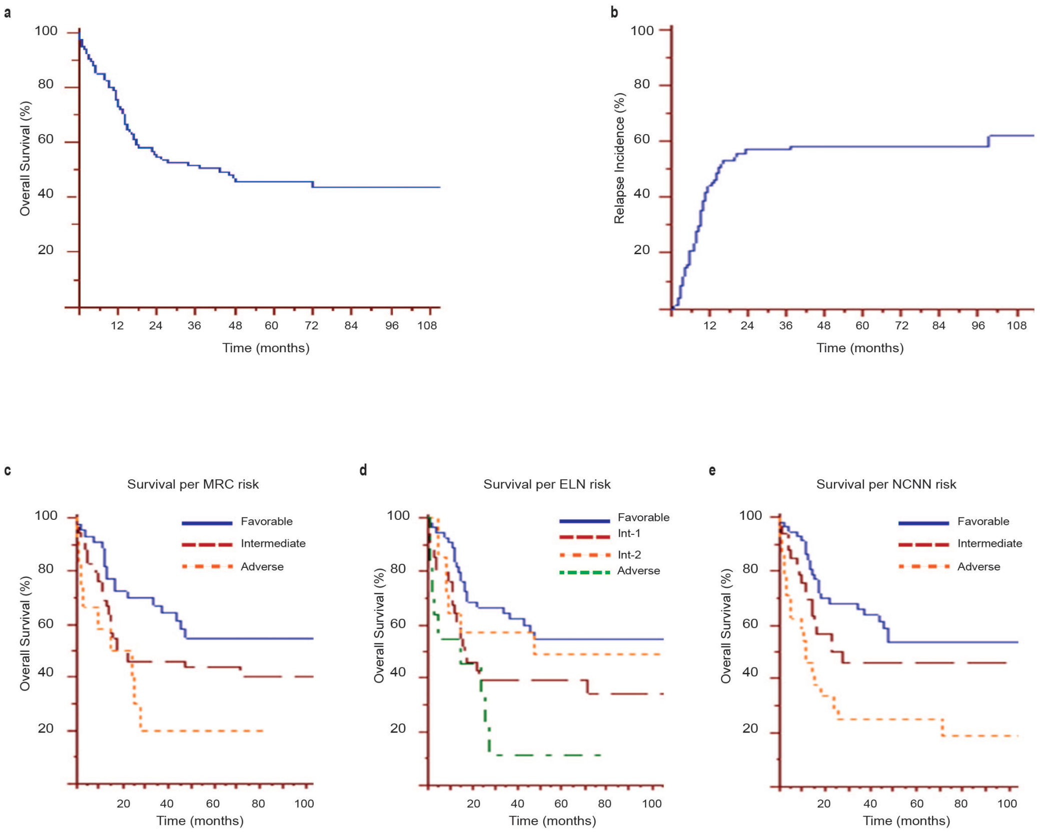

Supplementary Figure 2. Treatment course and outcome of study population.

a, b, Treatment schedule and outcome data were available for 116 out of 125 patients, assessed for response. Complete Response was obtained from 97 out of 116 (83.6%) patients. Primary refractory diseases and 5 infectious complication during post-chemotherapy aplasia accounted for the 19 patients who did not achieve CR.

The median follow-up time was 65.5 months based on the reverse Kaplan-Meier method. The estimated 5-year Overall Survival (**a**) and Relapse Incidence **(b**) resulted 45.8% and 58.1% respectively, with 37 patients alive in CR1 and 16 patients alive in second or subsequent CR. **c, d, e**, Kaplan-Meier plots showing five-years OS per risk classification. MRC risk classification: Favorable (54.9%), Intermediate (43.7%), Adverse (20.0%), *p* 0.0314. ELN risk classification: Favorable (54.7%), Intermediate-1 (39.3%), Intermediate-2 (49.0%), Adverse (11.4%), *p* 0.0133. NCNN risk classification: Favorable (53.6%), Intermediate (45.9%), Adverse (25.0%), *p* 0.0010.

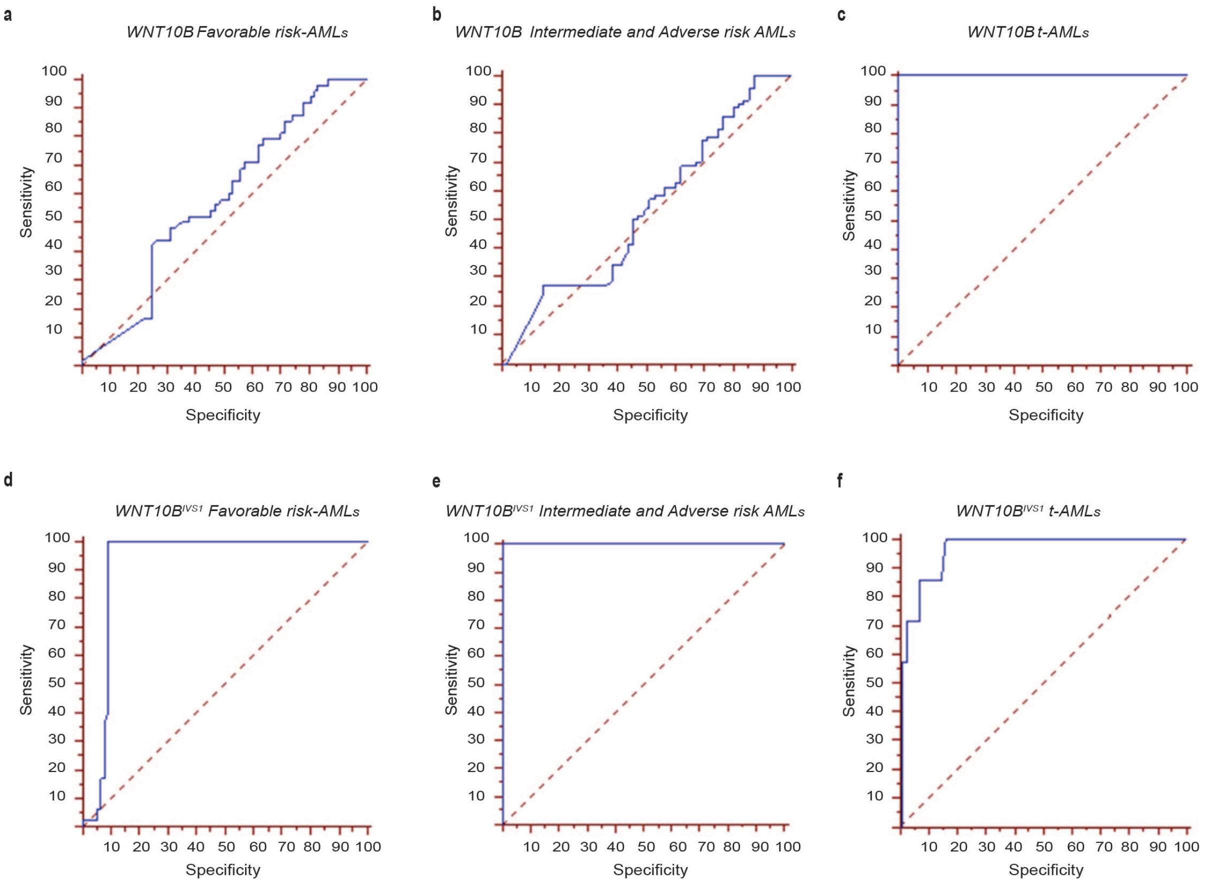

Supplementary Figure 3. Receiver Operating Characteristic (ROC) curve analysis.

Receiver Operating Characteristic (ROC) curve analysis of WNT10B and WNT10B^{IVS1} levels towards those three groups (favorable risk-AMLs, intermediate and adverse risk AMLs and t-AMLs). **a, b, c,** WNT10B analysis showed an optimal cut-point at 123 when test for therapy-related AMLs (AUC 1.0, sensitivity 100.0%, specificity 100.0%; p <0.0001), while no possible cut-off points were identified for favorable-risk (AUC 0.578, p 0.1432) or Intermediate and adverse risk AML-patients (AUC 0.533, p 0.5304). **d, e, f**, WNT10B^{IVS1} analysis showed an optimal cut-point at 221 when test for favorable-risk AMLs (AUC 0.918, sensitivity 100.0%, specificity 90.91%; p <0.0001), while a WNT10B^{IVS1} value lower then or equal to 32 is suggestive for t-AMLs (AUC 0.96, sensitivity 100.0%, specificity 83.76; p 0.0001). Similarly, a WNT10B^{IVS1} value higher then 221 is indicative of non-CBF/APL or non-therapy-related AMLs (AUC 1.0, sensitivity 100.0%, specificity 100.0%; p 0.0001).

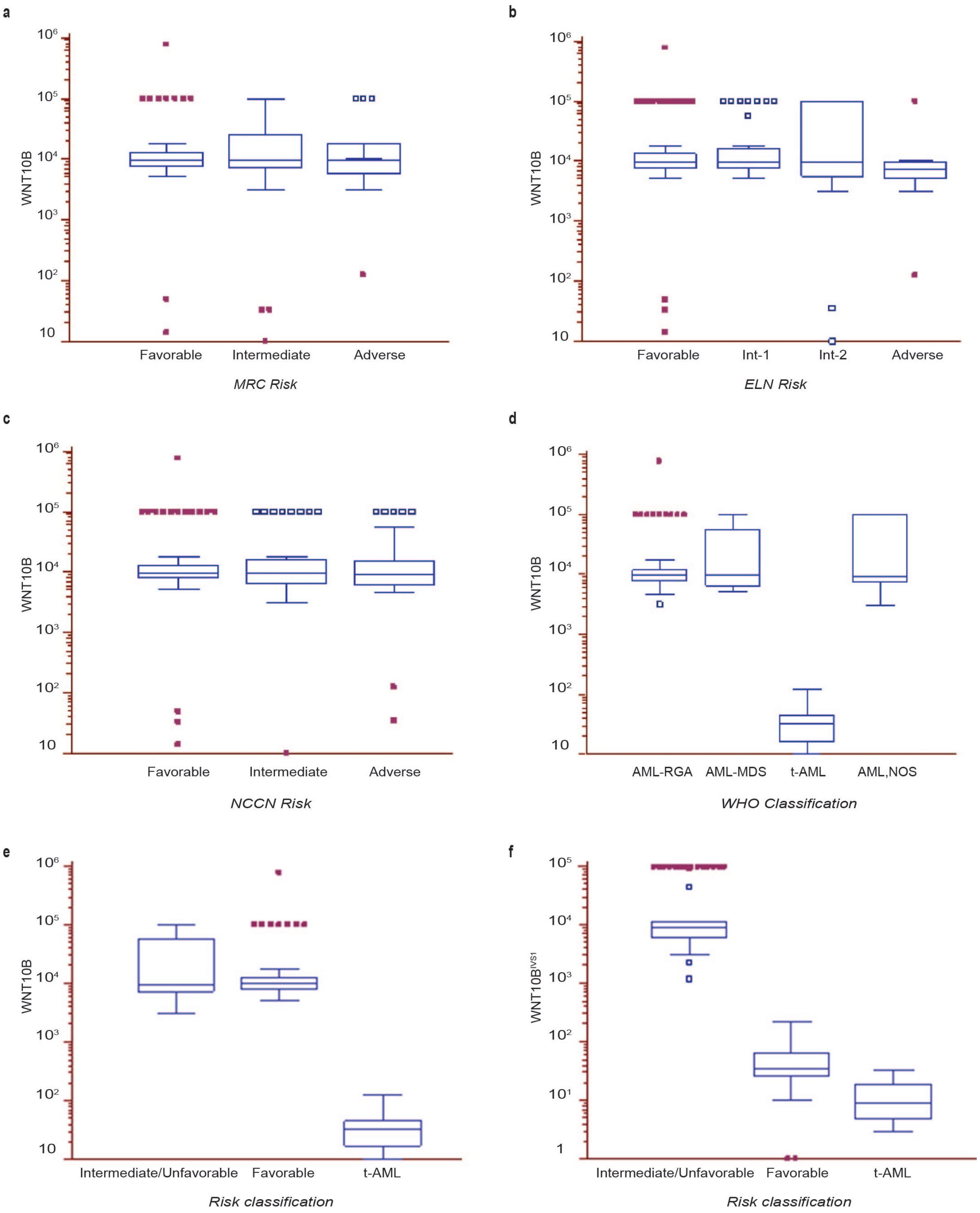

Supplementary Figure 4. Box-plot distribution of WNT10B/WNT10B^{IVS1} transcripts per AML classes.

a, b, c, No significative difference in distribution of WNT10B transcript levels according to the MRC, ELN and NCNN classification systems (*p* 0.6395, *p* 0.4295, and *p* 0.1786, respectively; Kruskal-Wallis test and comparative Mann-Whitney U-test). **d**, WNT10B transcript levels showed a statistical significative difference between the distinct WHO classes (*p* 0.0002; Kruskal-Wallis test). Comparative Mann-Whitney U-test confirmed a significative reduction of WNT10B levels in t-AML *versus* the other WHO groups (*p* <0.002). No statistical significance in WNT10B levels was found between patients with recurrent genetic abnormalities, myelodysplasia-related features or AML not otherwise specified. **e, f,** Kruskal-Wallis statistical test WNT-based classification of AMLs (*p* <0.0001): comparative Mann-Whitney U-test endorsed a reduction of WNT10B (**e**) in patients with t-AML when compared with favorable-risk (p <0.0001) and Intermediate-Adverse patients (p <0.0001). $WNT10B^{IVS1}$ (**f**) levels in favorable-risk patients were statistically different from t-AML patients (p 0.0012) and from Intermediate-Adverse AMLs (p <0.0001). Data represent mean values ± s.d. AML-RGA: AML with recurrent genetic abnormalities, AML-MDS: AML with myelodysplasia-related features, t-AML: therapy-related AML, AML,NOS: AML, not otherwise specified

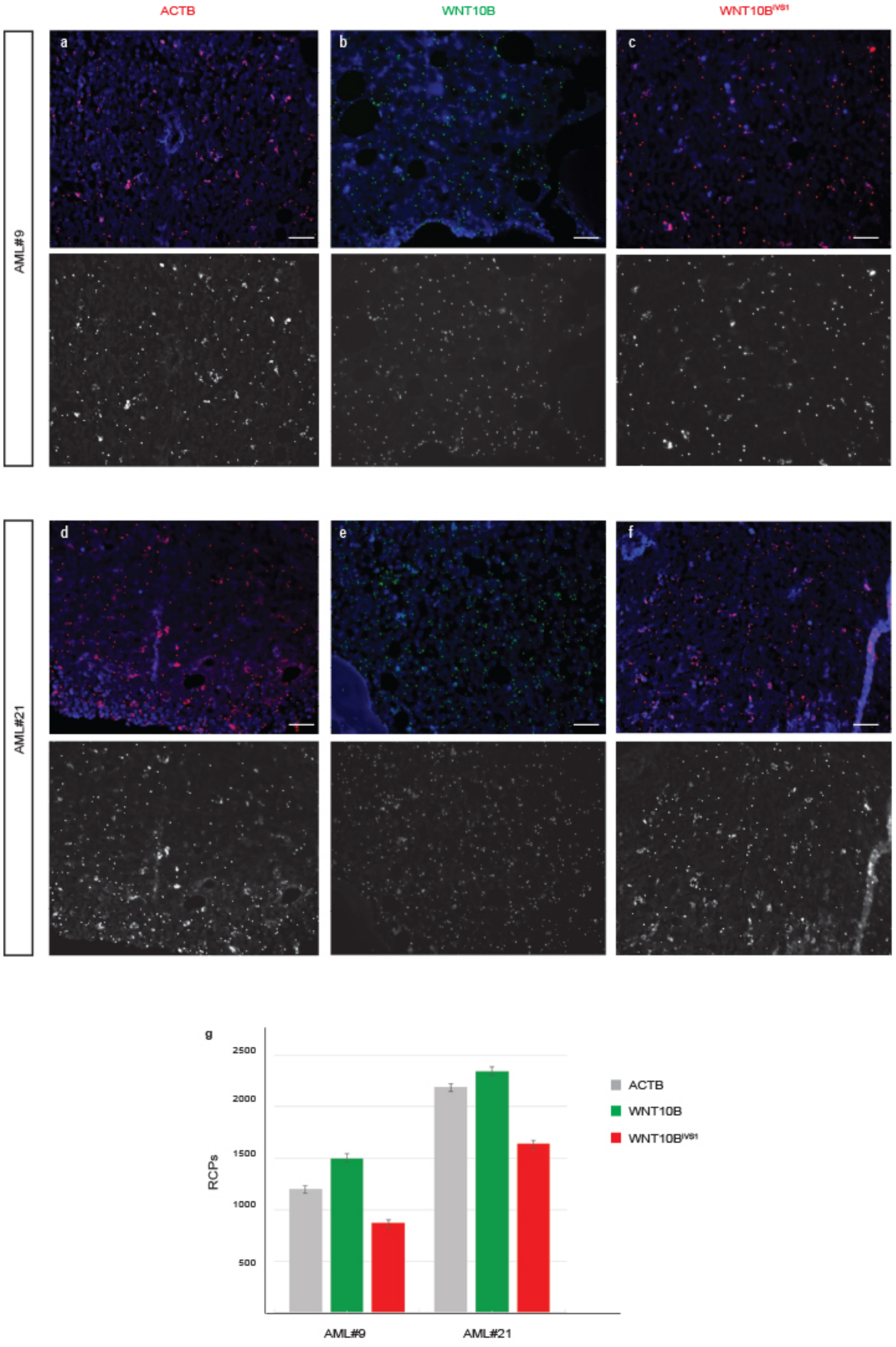

Supplementary Figure 5. mRNA *in situ* detection of WNT10B/WNT10B^{IVS1} transcripts on AML bone marrow sections.

In situ detection of β–actin (**a, d**), WNT10B (**b, e**) and WNT10B^{IVS1} (**c, f**) in FFPE tissue sections derived from AML#9 and AML#21 patients, using padlock probes and target-

primed RCA. Red RCPs (Cy3) represent β –actin and WNT10B^{IVS1} respectively and WNT10B RCPs are shown in green (Cy5). Exposure times: DAPI 450 ms, Cy3 500 ms and Cy5 600 ms. Merged images in the upper side of each panel, and the grey scale RCPs are represented in the lower panel. Scale bar 20 μm. **g,** Quantification of β–actin (grey), WNT10B (green) and WNT10B^{IVS1} (red) RCPs using CellProfiler software. Data represent mean values ± s.d.

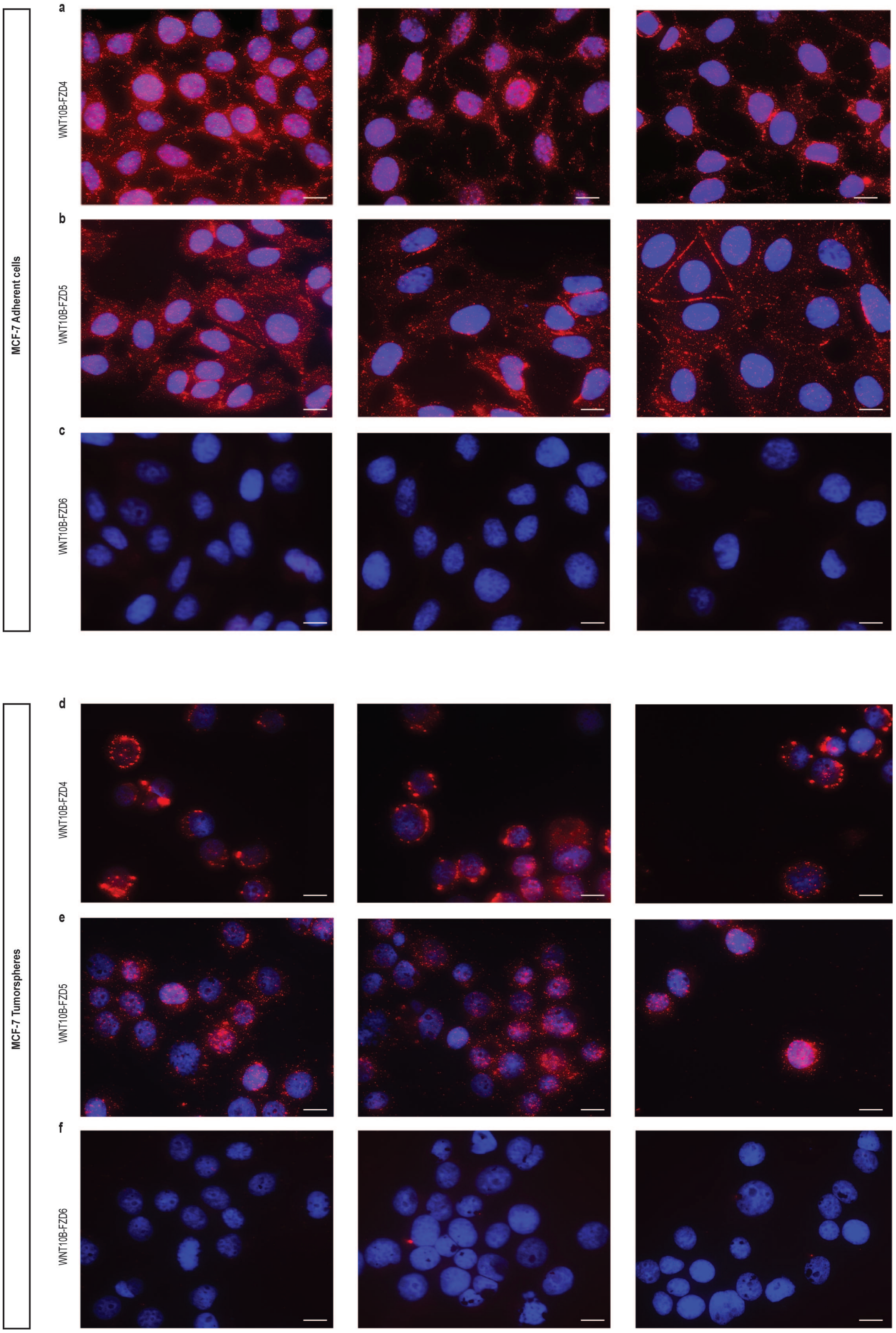
MCF-7 Adherent cells
a
WNT10B-FZD4
b
WNT10B-FZD5
c
WNT10B-FZD6
MCF-7 Tumorspheres
d
WNT10B-FZD4
e
WNT10B-FZD5
f
WNT10B-FZD6

Supplementary Figure 6. Detection of WNT10B-FZD4/5/6 complexes in MCF-7 cell line.

Detection of WNT10B-FZD4 (**a, d**), WNT10B-FZD5 (**b, e**) and WNT10B-FZD6 (**c, f**) interaction in MCF-7 adherent (upper panel) and tumorspheres (lower panel) cultured cells by *is*PLA. Three different Maximum-Intensity Projection (MIP) images per each interaction are represented. The RCPs are visualized using hybridization probes labeled with Alexa568 (red), and the nuclei are visualized using Hoechst (blue). Exposure times MCF-7 adherent cells: DAPI 350 ms, Cy3 200 ms. Exposure times MCF-7 tumospheres: DAPI 400 ms, Cy3 350 ms. Images were acquired with x40 magnification. Scale bars 10 μm.

Table S1. Characteristics of AML patients

Characteristics		
**Patients, no.**	125	
Median age, years (range)	51	(15 - 76)
No. men/no. women	67/58	
Median WBC, x 10^9/L (range)	16.3	(0.3 - 345)
Median Hb, g/dl (range)	8.8	(4.2 - 12.0)
Median PLT, x 10^9/L (range)	34.5	(7 - 296)
Median LDH, U/L (range)	792	(172 - 1500)
Median marrow blast, % (range)	75.0	(23 - 98)
Median peripheral blast, % (range)	58.0	(0 - 97)
Extramedullary disease, no. (%)	12	(9.6)
**Cytogenetic features**		
Without additional abnormalities, no. (%)	48	(38.4)
No. Abnormalities, no. (%)	13	(10.4)
Structure abnormalities, no. (%)	64	(51.2)
t(8;21)	18	
inv(16)/t(16;16)	24 *	
t(15;17) or variant	8	
**Mutational status**		
FLT3-ITD mutated cases, no. (%)	15/86	(17.4)
FLT3-TKD mutated cases, no. (%)	5/83	(6.0)
NPM1 mutated cases, no. (%)	20/46	(43.5)
Biallelic CEBPA mutated cases, no. (%)	2/11	(--)
KIT mutated cases, no. (%)	15/42	(35.7)
**Classification**		
de novo AML, no.	112	
AML with myelodysplasia-related features, no.	6	
Therapy-related AML, no.	7 *	

* Two patients presenting with CBF-AML inv(16)(p13q22) and one patient with normal karyotype AML with NPM1 mutation were clinically considered as to have a t-AML because of a prior exposure to cytotoxic agents with an adequate latency period. Analysis for WNT10B-WNT10B^{IVS1} succeded in recognize these t-AML, confirming the lacking of both WNT transcripts.

Table S2. t-AML prototypic cases

AML#280

Age, y / Sex	Tumor Histology	Elston Grading	TNM	Er, %	PgR, %	c-Erb-B2 (HER2)	MIB1, %	Therapies †
76 / F	Invasive ductal carcinoma, poorly-differentiated	G3	pT1cN0	70	60	2+	35-40	Radiation therapy (50 Gy)<br>Adjuvant endocrine therapy

†: treatment schedule:

- Adjuvant endocrine therapy: tamoxifene 20 mg die per 2 years, then anastrazole 1 mg die

Age, y / Sex	FAB	Cytogenetic	FLT3	NPM1	CEBPA	WBC, x10^9/L	MO Blast, %	Therapies †	Outcome
77 / F	M1	46,XX	ITD	Exon 12	wild type	148.0	90	“7+2” induction	D/prim ref dis

D, death; F, female; prim ref dis, MO, medullary; primary refractory disease

†: treatment schedule:

- “7+2” induction therapy: cytarabine 100 mg/mq in continuous infu sion per 7 days + idarubicin 12 mg/mq per 2 days

t-AML#6

Age, y / Sex	Tumor Histology	Elston Grading	TNM	Er, %	PgR, %	c-Erb-B2 (HER2)	MIB1, %	Therapies †
41 / F	Invasive ductal carcinoma, moderately-differentiated ‡	G2	pT1c(m) N1(mic)	15	70	1+	---	Adjuvant chemotherapy<br>Adjuvant endocrine therapy

‡: family history of breast cancer (mother, sister, maternal aunt): non mutated BRCA1 and BRCA2

†: treatment schedule:

- FAC chemoterapy: cyclophosphamide + doxorubicin + fluorouracil per 2 cycles
- Adjuvant endocrine therapy: anastrazole 1 mg die per 1 year, tamoxifene 20 mg die per 1 year, then exemestane 25 mg die

Age, y / Sex	FAB	Cytogenetic	FLT3	NPM1	CEBPA	WBC, $x10^9/L$	MO Blast, %	Therapies †	Outcome
44 / F	M0	Complex ‡	wild type	wild type	wild type	15.1	90	"7+3" induction<br>FLAIRG salvage<br>Clofa-AraC salvage	D/prim ref dis

D, death; F, female; MO, medullary; prim ref dis, primary refractory disease

‡: 45,XX,dic(5;14)(q10;p12),-7,del(12)(p13),+14,del(17)(q23),+der(14)del(14)(q13q22)

†: treatment schedule:

- "7+3" induction therapy: cytarabine 100 mg/mq in continuous infusion per 7 days + idarubicin 12 mg/mq per 3 days
- FLAIRG salvage: fludarabine 15 mg/mq bid per 5 days + cytarabine 1000 mg/mq bid per 5 days + idarubicin 10 mg/mq per 2 days
- Clofa-AraC salvage: clofarabine 22.5 mg/mq per 5 days + cytarabine 1000 mg/mq bid per 5 days

Table S3. Non Familiar Breast Cancer (NFBC) patients

# Patient	Age diagnosis	Tumor Histology	Elston Grading	TNM*	Er † (%)	PgR‡ (%)	c-Erb-B2 (HER2)	Ki67 (%)
**28**	49	Invasive Ductal Carcinoma, moderately differentiated	G2	na	10	10	na	15
**29**	59	Multiple Invasive Ductal Carcinoma, moderately differentiated	G2	pT2 (m);N0	10	10	na	25

*:TNM classification: T primary tumor site, N regional lymphnode involvement, M presence of distant metastatic spread.

†:Estrogen Receptors

‡: Progesterone Receptors

Table S4 .Oligonucleotide sequences

Zebrafish Primers	Sequences (5’-3’)
Zf-Wnt10b_P1	ATGGAGTTACCCCACAGACAG
Zf-Wnt10b_P2	GAGTGGGTTAATGTGTGCAAGTGA

Primers	Sequences (5’-3’)
WNT10B_P1	CTCCTCCAGCATGTCGAAGC
UNK_P2	GCCACGTCGACTAGTAC
WNT10B^{IVS1}_P3	CCTGAACCCGCATCAAGTCTC
WNT10B_P4	GCAGCACTAGTAAGCCCAG
WNT10B_P5	ATCTCATTGCTTAGAGCCCGAC
GAPDH_P6	ACCTGCCAAATATGATGACATC
GAPDH_P7	CAGTGTAGCCCAGGATGC
WNT10B_P8	C+A+G+G+C+CGGACAGCGTCAAGCACACG
ACTB_P9	C+TG+AC+CC+AT+GCCCACCATCACGCCC
WNT10B^{IVS1}_P10	CAAGGTAAGGCTGACCCTCAC
WNT10B_P11	GAGAAGGCTACACATCCCAGAG
FZD4_P12	GGCGGCATGTGTCTTTCAGT
FZD4_P13	GAATTTGCTGCAGTTCAGACTCTCT
FZD5_P14	CGCGAGCACAACCACATC
FZD5_P15	AGAAGTAGACCAGGAGGAAGACGAT
FZD6_P16	ACAAGCTGAAGGTCATTTCCAAA
FZD6_P17	GCTACTGCAGAAGTGCCATGAT
FZD8_P18	GCTCGGTCATCAAGCAACAG
FZD8_P19	ACGGTGTAGAGCACGGTGAAC
M13_P20	TTGTAAAACGACGGCCAGT
M13_P21	CAGGAAACAGCTATGACC

+=LNA-modified base.

TaqMan Probes	Sequences (5’-3’)
WNT10B_dd1	[6FAM] CACCCAAACCACTGGAGTCCTGATCG[BHQ1]
WNT10B^{IVS1}_dd2	[HEX] TCTCCCGTCCCGCAGGTCCTGATCG[BHQ1]

Padlock Probes	Sequences (5’-3’)
WNT10B_PP1	[Phos]-ACCGTGCCTGTCGGACCCTCCTCTATGATTACTGACCTAAGT<br>CGGAAGTACTACTCTCTTCTTCTTTTAGTGAAGCCCAGGCAACCCA
WNT10B^{IVS1}_PP2	[Phos]-AGTCTCCCGTCCCGCAGGTCTCCTCTATGATTACTGACCTA<br>TGCGTCTATTTAGTGGAGCCTCTTCTTTCTATTCCTGAACCCGCATCA
ACTB_PP3	[Phos]-GCCGGCTTCGCGGGCGACGATTCCTCTATGATTACTGACCTA<br>TGCGTCTATTTAGTGGAGCCTCTTCTTTACGGCGCCGGCATGTGCAAG

Detection Probes	Sequences (5’-3’)
DP1	[Cy3]-TGCGTCTATTTTAGTGGAGCC
DP2	[Cy5]-AGTCGGAAGTACTACTCTCT

PLA oligonucletides	Sequences (5’-3’)
Compaction Oligo_C1	AGAGAGTAGTACAGCAGCCGTAAAAGAGAGTAGTACAGCAGCCGT(UUU)
Detection Oligo_D1	CAGTGAATGCGAGTCCGTCT(UUU)
